# Podocyte injury damages podocytes in chimeric organoids

**DOI:** 10.1371/journal.pone.0337677

**Published:** 2026-08-31

**Authors:** Tomohiro Udagawa, Toshikazu Araoka, Kenji Osafune, Taiji Matsusaka

**Affiliations:** 1 Department of Physiology, Tokai University School of Medicine, Isehara, Japan; 2 Department of Pediatrics and Developmental Biology, Graduate School of Medical and Dental Sciences, Institute of Science Tokyo, Japan; 3 Center for iPS Cell Research and Application (CiRA), Kyoto University, Kyoto, Japan; Sri Ramachandra Institute of Higher Education and Research (Deemed to be University), INDIA

## Abstract

We have previously shown that injury to a subset of podocytes can trigger indirect damage in neighboring podocytes, but whether this phenomenon depends on direct intercellular interaction is unknown. To address this, kidney organoids were generated from nephron progenitor cells of two mouse lines: one expressing a receptor for a podocyte-specific immunotoxin and another expressing a tagged ribosomal protein. In chimeric organoids containing a mosaic of these podocyte types, immunotoxin exposure selectively injured the targeted podocytes and also induced indirect injury in adjacent, non-targeted podocytes. This was evidenced by reduced podocin staining and decreased expression of podocyte-specific genes in the non-targeted podocytes. The indirect injury was absent when organoids of each type were cultured separately but in close proximity, indicating that direct cell-to-cell contact within the same glomerular structure is required. These findings show that podocyte injury can propagate locally within kidney organoids, independent of glomerular filtration or other glomerular cell types, and suggest that local podocyte interactions may contribute to the progression of chronic kidney disease.

## Introduction

Podocyte injury is a critical initiating event in the development and progression of glomerulosclerosis [1–4]. In our previous study, we developed a transgenic mouse model in which podocytes express human CD25 (hCD25), allowing selective injury by the hCD25-targeted immunotoxin LMB2 [5]. In hCD25-expressing podocytes, LMB2 inhibits protein synthesis and induces apoptotic caspase activation [6]. Podocytes with activated caspase are immediately detached by filtration pressure before completing the full apoptosis process. The inhibition of protein synthesis, also seen in conditions like endoplasmic reticulum stress, is a key driver of human kidney disease progression [7–9].

Notably, a single injection of LMB2 results in progressive kidney injury over several weeks. This progression occurs despite the rapid clearance of the toxin and the elimination of caspase-activated podocytes within seven days. Similar progression has been observed in other models of podocyte injury [10]. In human and animal models, segmental sclerosis lesions are typically localized to a few regions per glomerulus despite widespread injurious stimuli. This local nature suggests that podocyte injury spreads locally and autonomously within the glomerulus [11,12].

To investigate this, we previously generated chimeric and mosaic mouse models in which only a subset of podocytes expresses hCD25 [13,14]. As expected, hCD25(+) podocytes were selectively injured following LMB2 injection. Surprisingly, adjacent hCD25-negative podocytes -which cannot bind LMB2- were also affected. These cells showed reduced nephrin and podocin and increased desmin expression. Ultimately, this led to global glomerulosclerosis. These findings indicate that injured podocytes can indirectly harm healthy neighboring podocytes in a self-amplifying cycle [12]. A similar indirect injury has been observed in other *in vivo* models [15,16]. However, this phenomenon could not be reproduced *in vitro* using traditional podocyte culture systems [14].

In conventional 2D cultures, podocytes rapidly lose expression of key differentiation markers such as nephrin and podocin. By contrast, kidney organoids generated from pluripotent stem cells or nephron progenitor cells (NPCs) contain more physiologically relevant, differentiated podocytes. To overcome the limitations of 2D culture, we utilized the culture-dependent purification (CDP) method developed by Li and Araoka [17]. This method allows for the purification and expansion of mouse NPCs, which differentiate into organoids containing physiologically relevant, glomerulus-like structures. Following brief stimulation with Wnt and FGF signals, progenitor cell aggregates differentiate into kidney organoids containing podocytes in glomerulus-like structures and tubular cells in tubular structures.

In the present study, NPCs were isolated from NEP25 mice and from mice lacking hCD25 to generate chimeric kidney organoids. Using this new *in vitro* system, we successfully isolated direct podocyte-podocyte communication from confounding *in vivo* factors, such as filtration pressure and other types of cells. We demonstrate for the first time *in vitro* that podocyte injury propagation is an autonomous, self-sustaining process driven by direct podocyte-to-podocyte interactions.

## Materials and Methods

### Mice

All animal experimental procedures were approved by the Animal Experimentation Committee of Tokai University School of Medicine and were conducted and reported in accordance with the Guide for the Care and Use of Laboratory Animals published by the U.S. National Institutes of Health and the ARRIVE guidelines (https://arriveguidelines.org).

Three transgenic mouse lines were used in this study, namely, NEP25 (carrying *Nphs1-hCD25*), Nephrin-Cre (*Nphs1-Cre*), and Ribotag (*Rpl22*^*tm1.1Psam*^). NEP25 [5] and Nephrin-Cre [18,19] lines were established in our laboratory and maintained in the C57BL/6N genetic background. Ribotag line (IMSR_JAX:011029) was obtained from the Jackson Laboratory and maintained by crossing the Nephrin-Cre line [18]. NEP25 mice expressed hCD25 selectively in podocytes, enabling the induction of selective podocyte injury by the administration of the hCD25-targeted immunotoxin, LMB2 (a gift of Ira Pastan) [5]. Nephrin-Cre/Ribotag mice expressed the ribosomal protein L22 (Rpl22) tagged with a hemagglutinin (HA) epitope specifically in podocytes [18]. To generate NPC lines, mice carrying the combinations of *Nphs1-hCD25* (0 or 1 copy), *Nphs1-Cre* (0 or 1 copy), and *Rpl22*^*tm1.1Psam*^ (2 copies) were inter-crossed, and embryos were harvested at embryonic day 12.5. The primers used for genotyping are listed in Table A in S1 Table.

### Organoid Culture

NPC lines were established using the CDP method with modifications [6,17]. In brief, the kidneys were isolated from each embryo, and 1 × 10⁴ cells were seeded in NPSR medium on laminin-coated 12-well plates. The FGF-2 and 2-mercaptoethanol concentrations were increased to 300 ng/mL and 0.1 mM, respectively.

Simultaneously, the presence of *Nphs1-Cre* and *Nphs1-hCD25* transgenes was determined by real-time PCR analysis of genomic DNA from embryonic tail biopsies. Cells derived from embryos carrying *Nphs1-hCD25* or *Nphs1-Cre* were selected for further purification. After 4 days of culture, floating cell aggregates were collected, dissociated, and re-cultured in 96-well U-bottom low-attachment plates with NPSR medium. Aggregates were passaged every 4 days. Cell lines that are capable of sustained proliferation formed spherical aggregates over 10 passages. The cells from passages 4–16 were used for organoid generation.

For differentiation, aggregates with a diameter of ~1 mm were transferred to Transwell membranes and cultured in KR5-CF medium containing 4.5 μM CHIR99021 and 300 ng/mL FGF-2 for 2 days and then in KR5 medium alone. LMB2 (20 nM) was added on day 6, and the culture was continued for 2–4 days. Then, the organoids were subjected to histological and polysome analyses. In selected experiments, organoids were co-treated with 100 μM carbenoxolone disodium (CBX, gap junction inhibitor), 1 μM SAR7334 (TRPC6 inhibitor), 1 μM A83-01 (TGF-β receptor inhibitor), or 2 μM IKK-16 (NF-κB inhibitor) alongside LMB2.

### Histological analysis

Organoids were fixed in 4% paraformaldehyde, embedded in paraffin, and sectioned at 2 μm for PAS staining and immunohistochemistry. Some organoids were fixed, permeabilized with 0.1% Triton X-100 in PBS, and stained with fluorescent markers for WT1 and Lotus tetragonolobus lectin (LTL). The details of the primary antibodies and lectins are provided in Table B in S1 Table. In addition, CanGet signal amplification solutions (Toyobo, Osaka, Japan) were used to detect HA, WT1, podocalyxin, and podocin.

Brightfield images were acquired using an Axioplan 2 microscope (Zeiss), and immunofluorescent images were obtained using a BZ-X710 microscope (Keyence).

For the experiments with LMB2 treatment, podocin and HA staining in the adjacent sections were photographed and quantified using Fiji software. The integrated intensity of podocin was normalized to either the HA-positive area or the total organoid area for each organoid.

### RNA Analysis

Approximately 14–16 organoids were harvested after treatment with TrypLE containing cycloheximide (100 μg/mL). Then, the samples were homogenized in 220 μL of lysis buffer (50 mM Tris [pH 7.4], 100 mM KCl, 12 mM MgCl_2_, 1% Nonidet P-40, 1 mM DTT, 200 U/mL RNasin, 1 mg/mL heparin, 100 μg/mL cycloheximide, and 1% protease inhibitor cocktail) and vortexed for 1 min. After centrifugation (10,000 rpm, 10 min, 4°C), HA-tagged ribosomes were immunoprecipitated (IP) using an anti-HA.11 antibody (clone 16B12) (S7 Fig) as previously described [18]. Briefly, 2.5 μg of anti-HA.11 epitope tag antibody was added to the supernatant, and the sample was rotated for 4 hours at 4°C. 100 μL of Dynabeads Protein G (Thermo Fisher) was then added to the samples, and they were rotated overnight at 4°C. On the following day, the samples were placed on a magnet on ice, and the supernatant was separated. The remaining pellet was washed 3 times for 10 minutes in a high-salt buffer (50 mM Tris, pH 7.4, 300 mM KCl, 12 mM MgCl_2_, 1% Nonidet P-40, 1 mM DTT, 100 μg/mL cycloheximide). RNA was extracted from IP polysomes using the RNeasy Micro Kit (QIAGEN), and RNA was extracted from unbound fractions using the RNeasy Mini Kit (QIAGEN). The columns of the RNeasy Micro Kit were eluted with 40 μL of water, and ethanol precipitated. Quantitative RT-PCR was performed using the ΔΔCT method, with *Actb* as the internal control. The primer and probe information is shown in Tables C and D in S1 Table.

### Statistical analysis

Log-transformed RNA ratios (IP polysomes vs. the supernatant, and IP with LMB2 vs. IP without LMB2) were analyzed by one-sample t-tests, with comparison to log (1) = 0. Holm’s method was used to adjust the *p*-values. Relative *hCD25* mRNA levels were compared using unpaired t-tests. All statistical analyses were performed using EZR version 4.5.0, and *p*-value < 0.05 was considered statistically significant. Data were logarithmically transformed to satisfy the assumption of normality for the one-sample t-test. The robustness of the statistical significance was confirmed by alternative transformation methods (square-root or reciprocal) where appropriate.

### AI tools

ChatGPT-4 was used to check and correct grammatical errors in the manuscript.

## Results

### Generation of Kidney Organoids from NEP25 and Ribotag Mice

**Fig 1:**
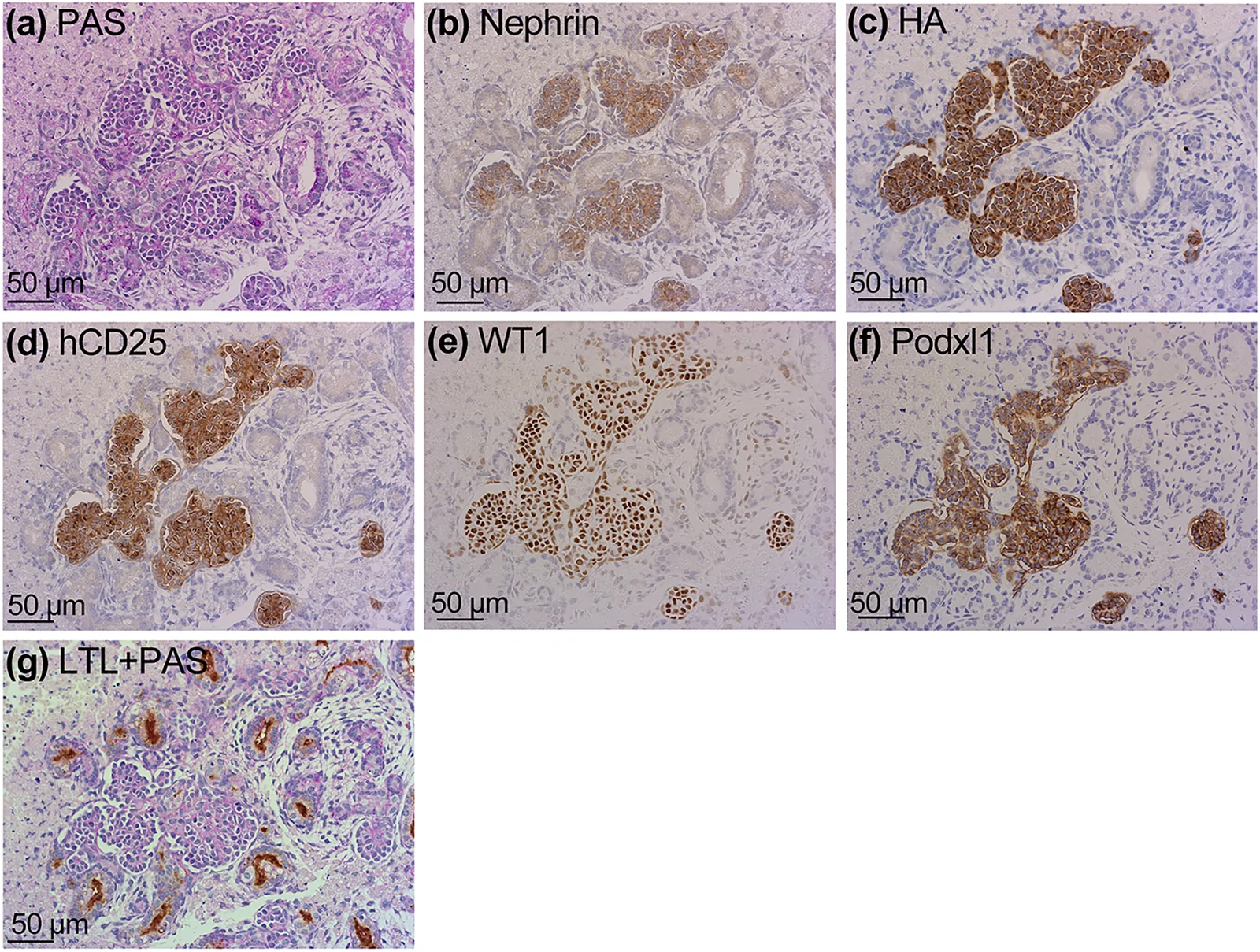
Characterization of kidney organoids derived from NEP25/RiboTag mice. Kidney organoids were generated from NEP25/Ribotag nephron progenitor cells. (a–f) Glomerulus-like clusters visualized by PAS staining (a) and immunostaining for podocyte markers: nephrin (b), HA (c), hCD25 (d), WT1 (e), and podocalyxin-1 (f). (g) Tubular structures identified by LTL staining. Images (a–g) represent equivalent visual fields from serial sections.

**Fig 2:**
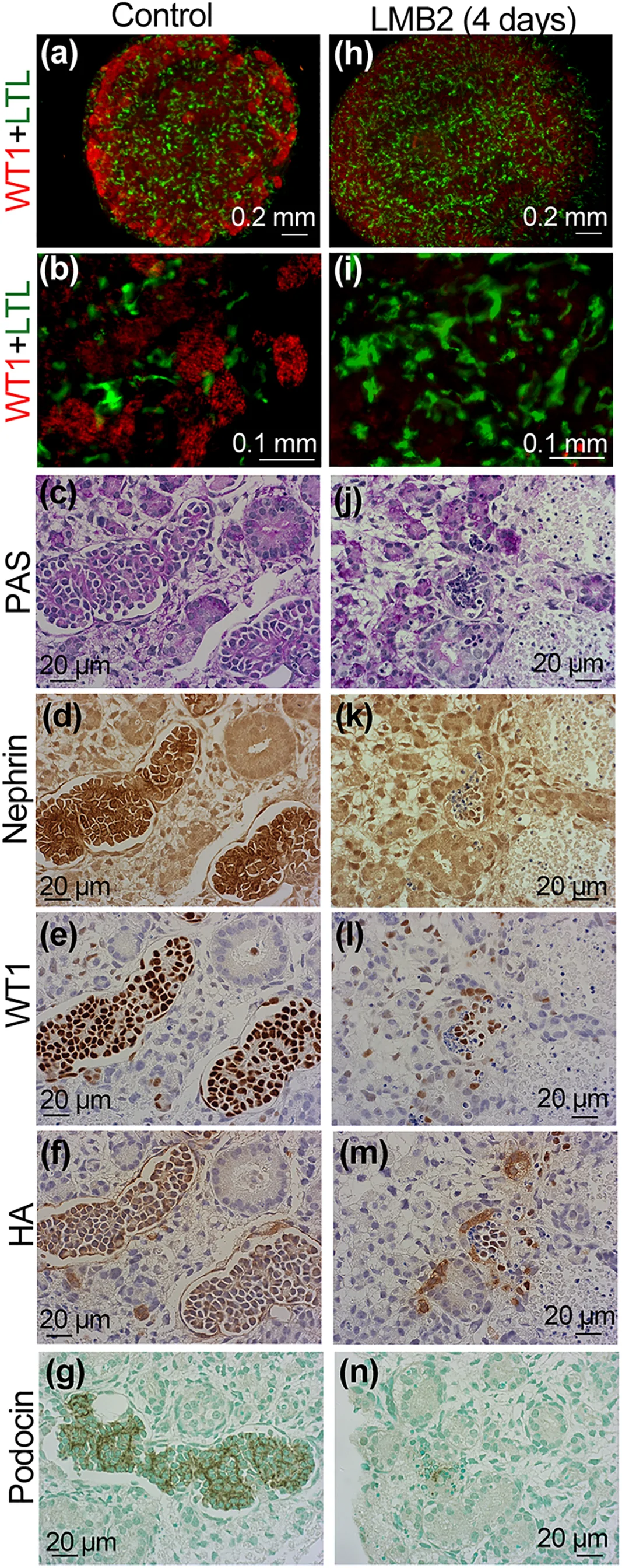
Selective podocyte injury induced by LMB2 in NEP25/RiboTag organoids. (a, b) Expression of WT1 and LTL in control organoids. (h, i) Effects of LMB2 treatment (4 days); WT1 expression is markedly reduced, while LTL remains unaffected. (c–g, j–n) Higher magnification of podocyte clusters in control (c–g) and LMB2-treated (j–n) organoids stained for nephrin, WT1, HA, and podocin. LMB2 treatment causes the loss of cluster morphology and podocyte marker expression, whereas LTL-positive proximal tubule structures remain unaffected, demonstrating the cell-type specificity of the immunotoxin. Panels (c–f) and (j–m) show equivalent visual fields from serial sections. Quantification of podocin staining intensity is provided in Table S2A in S2 Table.

Using the CDP method, NPCs were obtained from three types of transgenic embryos, namely, (1) NEP25 (carrying *Nphs1-hCD25*), (2) Ribotag (*Nphs1-Cre*), and (3) NEP25/Ribotag (*Nphs1-hCD25/Nphs1-Cre*). The NPC aggregates were cultured on Transwell membranes and exposed to CHIR99021 and FGF-2 to initiate differentiation. Although these organoids did not fully recapitulate the kidney’s architecture because of the absence of endothelial, interstitial, and ureteric bud-derived cells, they developed glomerulus-like and tubular structures. Immunostaining confirmed the presence of differentiated podocytes and proximal tubular cells, as evidenced by the expression of WT1, nephrin, podocalyxin, podocin, and LTL (Fig 1 and Fig 2). In addition, NEP25/Ribotag organoids co-expressed hCD25 and HA in podocytes, mirroring the parental transgenic mice.

### LMB2-induced Selective Podocyte Injury in NEP25 Organoids

Treatment of NEP25 and NEP25/Ribotag organoids with LMB2 resulted in podocyte-specific injury. Fluorescent imaging revealed reduced WT1 staining and preserved LTL staining 4 days after treatment (Fig. 2). During the 4-day treatment, the medium was changed, which washed away cell debris. In NEP25/Ribotag organoids, PAS and immunostaining showed the loss of podocyte clusters, with only scattered HA-positive cells between tubular structures. The expression level of WT1, nephrin, and podocin was markedly reduced, while megalin and LTL signals persisted (Fig. S1). Two days after the treatment, residual HA-positive clusters remained WT1 positive, but they showed decreased nephrin and podocin staining (S2 Fig).

**Fig 3:**
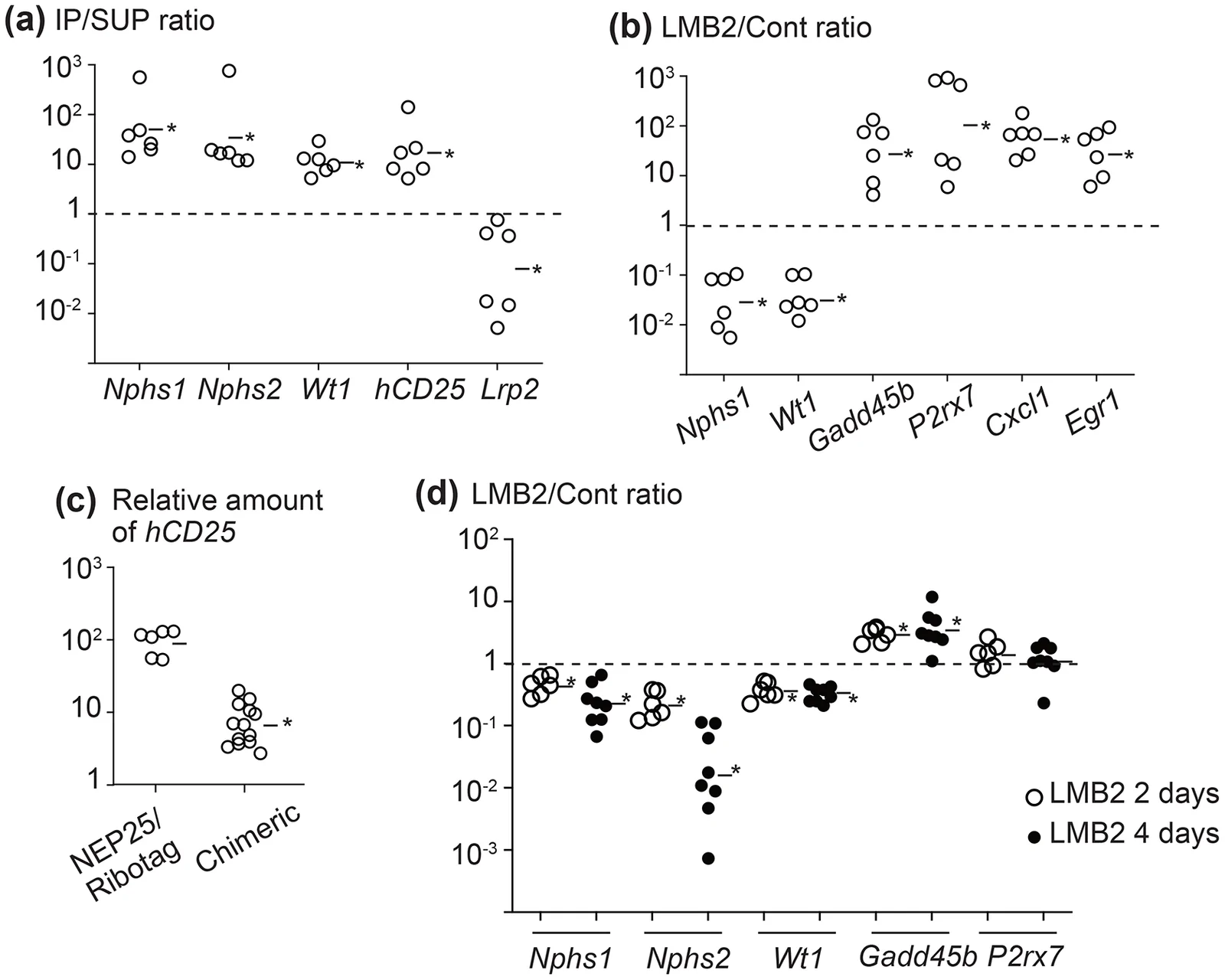
Quantitative RNA analysis of podocyte injury in organoids. (a) Enrichment of podocyte-specific polysomal RNA (IP) relative to total organoid RNA (SUP) in NEP25/RiboTag organoids (n = 6). (b) Fold-change in polysomal RNA expression following LMB2 treatment in NEP25/RiboTag organoids (n = 6). (c) Comparison of hCD25 mRNA abundance in IP samples between chimeric (n = 13) and NEP25/RiboTag (n = 6) organoids. (d) Expression changes in polysomal RNA specifically from hCD25(−) podocytes within chimeric organoids after LMB2 treatment. *Nphs2* was undetectable in all LMB2-treated samples, and not shown in the graph. Bars represent geometric means. Statistical significance was determined by one-sample t-test (a, b, d) or unpaired t-test (c); *p < 0.05.

HA immunoprecipitation enabled the isolation of podocyte-specific polysomes in NEP25/Ribotag organoids. In untreated organoids, *Nphs1*, *Nphs2*, *Wt1*, and *hCD25* mRNAs were enriched in IP samples, whereas *Lrp2* (megalin) was diluted (Fig 3). LMB2-treated organoids showed undetectable *Nphs2*, reduced *Nphs1* and *Wt1* expression levels, and increased injury-related transcript expression, including *Cxcl1*, *Gadd45b*, *Egr1*, and *P2rx7*, which is consistent with injured podocyte profiles in NEP25 mice [18](Fig 3b). Of note, suppression of *Nphs1* and *Nphs2* mRNAs was more pronounced after 4-day LMB2 treatment than 2-day treatment, suggesting a time-dependent accumulation of the indirect injury signal.

### Podocyte injury propagates in chimeric organoids

**Fig 4:**
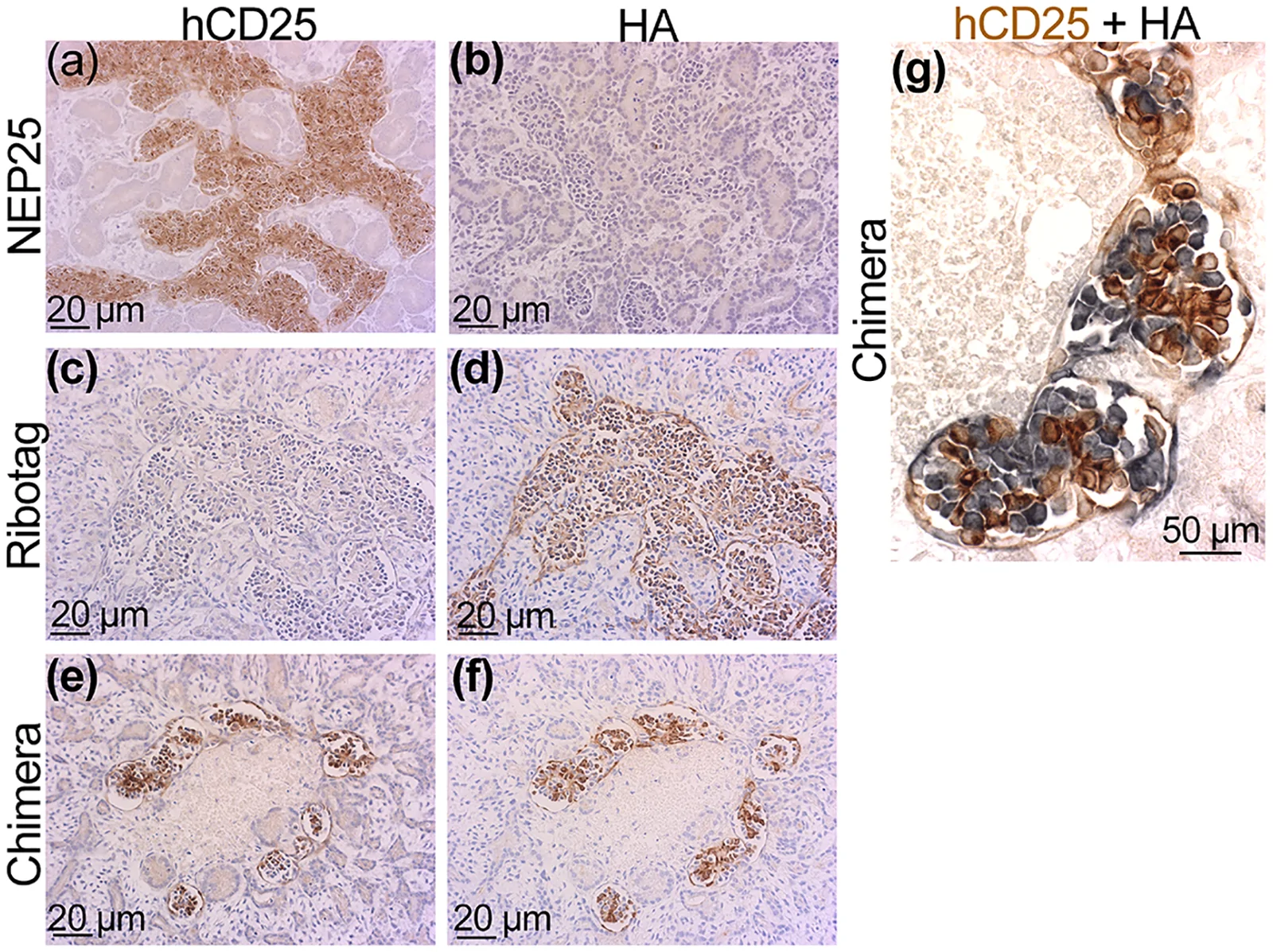
Validation of chimeric kidney organoids. Immunostaining for hCD25 and HA in (a, b) NEP25 organoids, (c, d) RiboTag organoids, and (e, f) chimeric organoids. (g) High-magnification image of a chimeric cluster showing mutually exclusive expression of hCD25 (brown) and HA (gray), confirming successful mosaicism of different podocyte genotypes.

**Fig 5:**
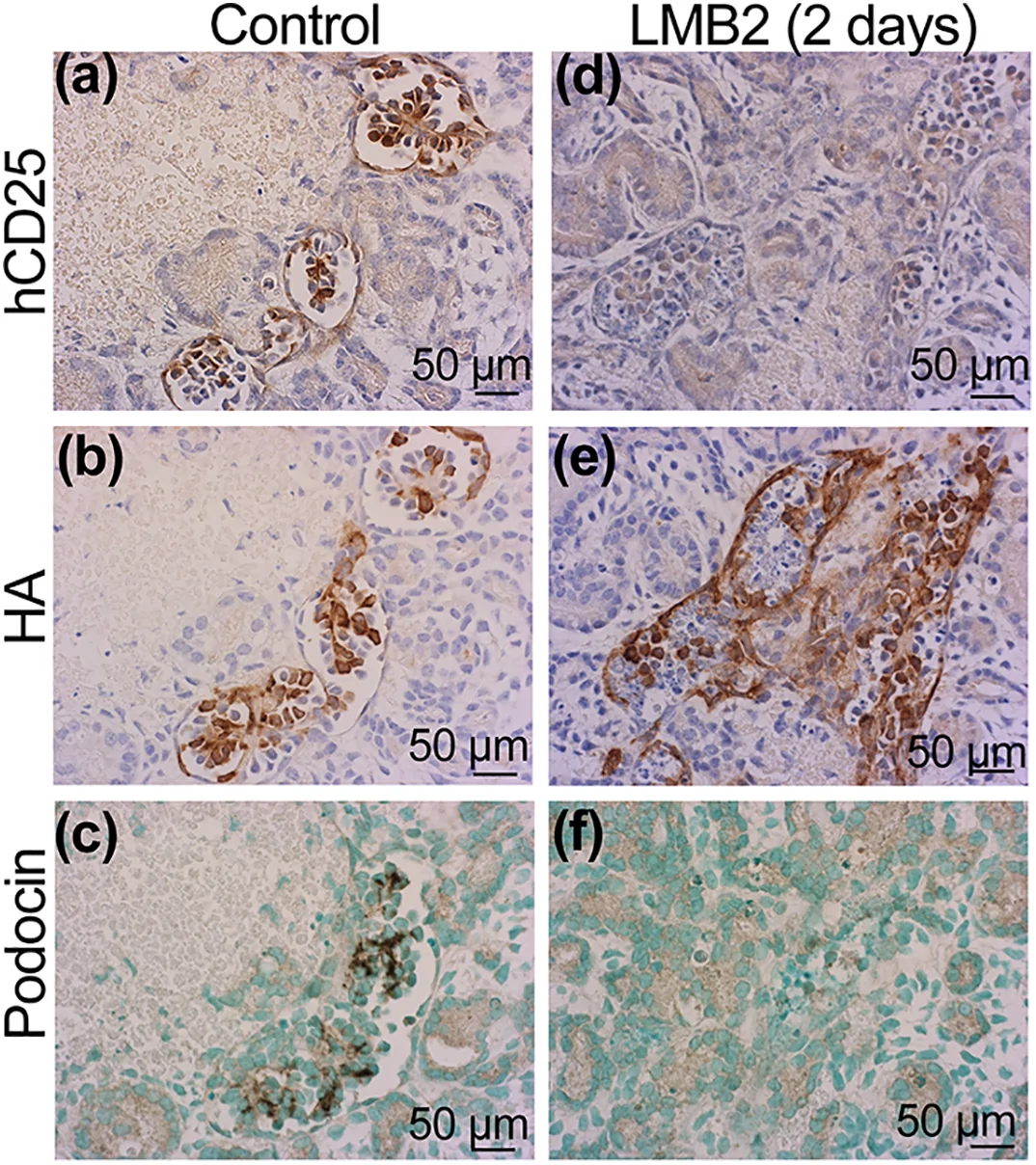
Propagation of podocyte injury (bystander effect) in chimeric organoids. (a–c) Robust expression of hCD25, HA, and podocin in untreated control chimeric organoids. (d–f) Chimeric organoids after LMB2 treatment. Note the complete loss of hCD25(+) podocytes (d) and the indirect reduction of podocin staining (f) in the persisting HA(+) podocytes (e), visually demonstrating the propagation of injury to non-targeted neighboring cells within the same glomerular-like structure. Panels (a–c) and (d–f) represent equivalent fields of serial sections. Quantified data for podocin intensity are shown in Table S2B in S2 Table.

Chimeric organoids were generated by combining NEP25 and Ribotag NPCs at a 1:1 ratio to assess the potential intercellular propagation of podocyte injury. Podocytes in these organoids expressed either hCD25 or HA but not both (Fig 4). Two days after the LMB2 treatment, the expression of hCD25 was lost, but HA staining persisted. Despite the survival of HA-positive podocytes, podocin staining was nearly absent (Fig 5).

By contrast, the coculture of separate NEP25 and Ribotag organoids on the same Transwell showed that podocin loss occurred only in NEP25 organoids (S3 Fig), indicating that the indirect effect requires close cell-cell contact.

**Fig 6:**
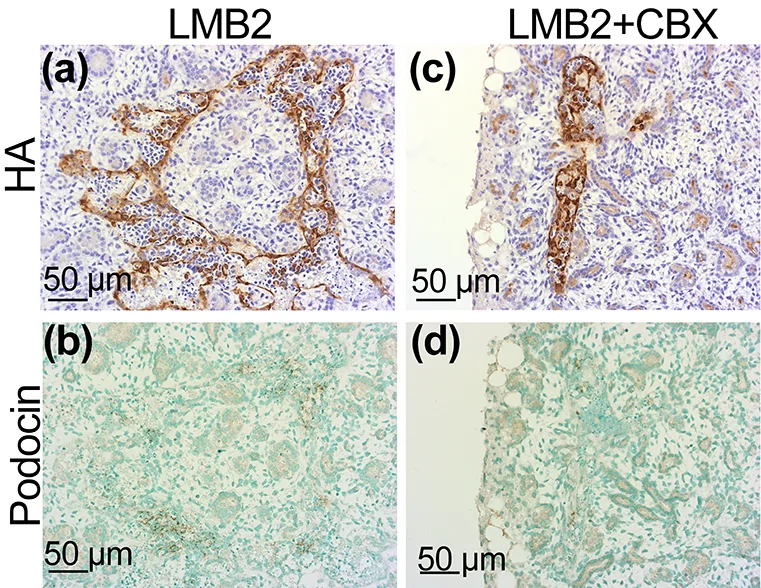
Effect of gap junction inhibition on indirect podocyte injury. Chimeric organoids were treated with LMB2 alone (a, b) or co-treated with the gap junction inhibitor carbenoxolone (CBX) (c, d). (a, c) HA staining confirms the presence of bystander podocytes. (b, d) CBX treatment fails to rescue the loss of podocin expression induced by LMB2, suggesting gap junctions are not the primary mediators of this injury propagation. (a–b) and (c–d) show equivalent visual fields from serial sections. Data quantification is provided in Table S2C in S2 Table.

In chimeric organoids, HA-immunoprecipitated RNA represents transcripts from hCD25-negative (LMB2-unexposed) podocytes. The mRNA levels of *hCD25* were significantly lower in chimeric IP samples than in NEP25/Ribotag organoids (Fig 3c). Following LMB2 treatment, these indirectly affected podocytes displayed reduced *Nphs1*, *Nphs2*, and *Wt1* expression levels and increased *Gadd45b* expression level, which is consistent with indirectly injured podocyte profiles in chimeric mice [13](Fig 3d). Because it is frequently reported that gap junctions mediate bystander killing in other tissues by transferring cytotoxic molecules, we hypothesized that intercellular signaling through gap junctions might mediate the indirect injury. RT-PCR revealed a high expression level of *Gja3* (Cx46), *Gja5* (Cx40), and *Gjc1* (Cx45) but a low expression level of *Gja1* (Cx43) in the podocytes of mosaic mice and organoids (S5 Fig). Treatment with CBX, which is a gap junction inhibitor, failed to prevent the loss of podocin in chimeric organoids (Fig. 6). Additional treatments with SAR7334 (TRPC6 inhibitor), A83-01 (TGF-β inhibitor), or IKK-16 (NF-κB inhibitor) also failed to block the injury (S4 Fig).

## Discussion

We hypothesized that indirect podocyte injury occurs in kidney organoids. In chimeric organoids containing both immunotoxin-sensitive and -resistant podocytes, we found that primary injury to a subset of podocytes led to indirect damage in neighbors. We confirmed this through podocin staining and quantitative polysome analysis (14–16 organoids per data point). These findings indicate that podocyte injury can propagate independently of glomerular filtration or interaction with other glomerular cell types.

Indirect injury, which is also referred to as bystander killing, is well recognized in nervous tissue damage models [20]. However, our initial attempt to recapitulate this effect in conventionally cultured podocytes was unsuccessful. Factors unique to the *in vivo* environment, such as the physical forces of glomerular filtration, macromolecular leakage into the Bowman’s space, or interactions with other types of glomerular cells, may contribute to the propagation of podocyte injury. Previous ultrastructural analysis of NEP25 mice revealed that glomerular filtration triggers the detachment of podocyte clusters from the glomerular basement membrane. This detachment was preceded by the expansion of the subpodocyte space, forming pseudocysts [21]. These findings indicate that initially uninjured podocytes adjacent to damaged ones may also be lifted and detached by the pseudocyst. Such hemodynamic forces may contribute to indirect podocyte damage because glomerular filtration exacerbates podocyte injury [22].

Although the involvement of these *in vivo* factors cannot be excluded, this study showed that indirect podocyte injury can occur independently of glomerular filtration. Our findings also indicate that other glomerular cell types, namely endothelial and mesangial cells, are not necessary for this process. Within two days after LMB2 treatment, podocin staining disappeared in hCD25-negative podocytes of chimeric organoids, but not in hCD25-negative organoids co-cultured with hCD25-positive organoids. These observations indicate that direct cell-cell communication mediates the indirect podocyte injury. Such communication may be mediated via the interaction of membrane proteins, paracrine factors, extracellular vesicles, gap junctions, or tunneling nanotubes (TNTs) [23].

Gap junctions facilitate the intercellular transfer of small molecules, including ions and reactive oxygen species. Notably, they also transport 2’ 3’-cGAMP, the second messenger for the STING pathway [23,24]. Connexins (Cxs) are gap junction proteins that facilitate intercellular organelle transfer and contribute to TNT formation [24,25]. Many studies have implicated the role of Cxs in bystander killing in radiation-induced and ischemic brain injury models [20,23,26]. Injured podocytes have been reported to express Cx43 and Cx45 [27,28], and our previous microarray analyses revealed that mouse podocytes express *Gja3* (encoding Cx46) and *Gja5* (Cx40) [18]. Our quantitative RT-PCR analysis revealed that *Gja3* was abundantly expressed at baseline and downregulated following LMB2 treatment in the podocytes of mosaic mice and organoids. The expression of *Gja1* (Cx43) was minimal; however, low levels of Gja5 and Gjc1 (Cx45) were detected after injury in the podocytes of mosaic mice (S5 Fig). To test their role in our model, we treated chimeric organoids with the inhibitor carbenoxolone [20]. Unlike previous findings in neural tissue, carbenoxolone did not prevent podocin loss in indirectly injured podocytes. While these pharmacological results suggest that gap junctions may not be the primary mediators of indirect injury in this model, further genetic validation would be required to definitively exclude their contribution.

TNTs represent another direct way of intercellular communication. These membranous structures can bridge distant cells and transfer a wide range of cargos, from ions to organelles [29]. Barutta et al. reported that podocytes express M-Sec (*Tnfaip2*), which is a key regulator of TNT formation [30,31]. *In vitro*, Adriamycin-exposed podocytes formed TNTs in a *Tnfaip2*-dependent manner. Furthermore, *Tnfaip2*-knockout mice with a BALB/c background spontaneously developed FSGS with associated mitochondrial dysfunction. In C57BL/6 mice, *Tnfaip2* deficiency caused no baseline renal abnormalities. However, it exaggerated podocyte injury, lysosomal alterations, and impaired autophagy following streptozotocin-induced diabetes [32]. These findings indicated that TNTs may play a protective role by transferring organelles from healthy to injured podocytes. However, given their bidirectional nature, TNTs could mediate injury propagation. In our current model, LMB2 directly injured approximately 50% of podocytes in chimeric organoids. Under such conditions, it is conceivable that injury signals may be transmitted from hCD25-positive to hCD25-negative podocytes via TNTs. RT-PCR analysis revealed that *Tnfaip2* mRNA was expressed in the podocytes of mosaic mice and organoids. LMB2 downregulated the expression level of *Tnfaip2* in hCD25-positive podocytes from NEP25/Ribotag organoids but not in those from mosaic mice (S6 Fig). Direct assessment of TNT involvement remains an objective for future studies.

Despite the remarkable results, this study has limitations. First, the molecular mechanism underlying the indirect injury could not be elucidated. It should be noted that our results using specific inhibitors, such as CBX for gap junction blockade, only partially rule out these signaling pathways. As these experiments do not constitute a comprehensive mechanistic screen, further studies are required to fully elucidate the underlying pathways. Second, the direct injury caused by LMB2 may not generally represent podocyte injury in actual kidney diseases. Third, our organoids are generated exclusively from nephron progenitor cells, thereby lacking components derived from interstitial progenitors and ureteric buds. Consequently, the tissue architecture of these organoids differs from that of the *in vivo* counterpart. While the absence of endothelial and mesangial cells allowed us to isolate and prove that they are not required for injury propagation, in native glomerular diseases, these cell types likely modulate the autonomous podocyte pathways described here.

Our findings indicate that podocytes directly injured by LMB2 can induce indirect damage to neighboring, initially intact podocytes within chimeric organoids. Importantly, this propagation occurs even in the absence of glomerular filtration or other glomerular cell types. Therefore, direct cell-cell communication plays a central role in the intraglomerular spread of injury. Further investigation is necessary to identify the molecular mechanism underlying this indirect podocyte injury in the chimeric organoid and mosaic mouse models. Such mechanisms may also contribute to injury amplification in various glomerular diseases, forming a vicious cycle that leads to global glomerulosclerosis.

## Supporting information

S1 Table Supplementary Tables A-D. (DOCX)

S1 Fig Tubular markers in NEP25/Ribotag organoids. (PDF)

S2 Fig NEP25/Ribotag organoids treated with LMB2 treatment for 2 days. (PDF)

S3 Fig Podocyte injury does not propagate between organoids. (PDF)

S4 Fig Effects of inhibitors on indirect podocin suppression in chimeric organoids. (PDF)

S5 Fig Connexin mRNA expression in mosaic mice and organoids. (PDF)

S6 Fig Tnfaip2 mRNA expression in mosaic mice and organoids. (PDF)

S7 Fig Schema showing polysome analysis of organoids. (PDF)

S2 Table Quantification of podocin staining. (XLSX)

S3 Table Ct values of RNA analysis. (XLSX)
