# Supplementary material for: Podocyte injury damages podocytes in chimeric organoids
Source: PLoS One. 2026 Aug 31;21(8):e0337677. doi: 10.1371/journal.pone.0337677 (PMC13528954; doi:10.1371/journal.pone.0337677)
Supplement: Supplementary file 1 [file pone.0337677.s002.pdf]

S1 Figure

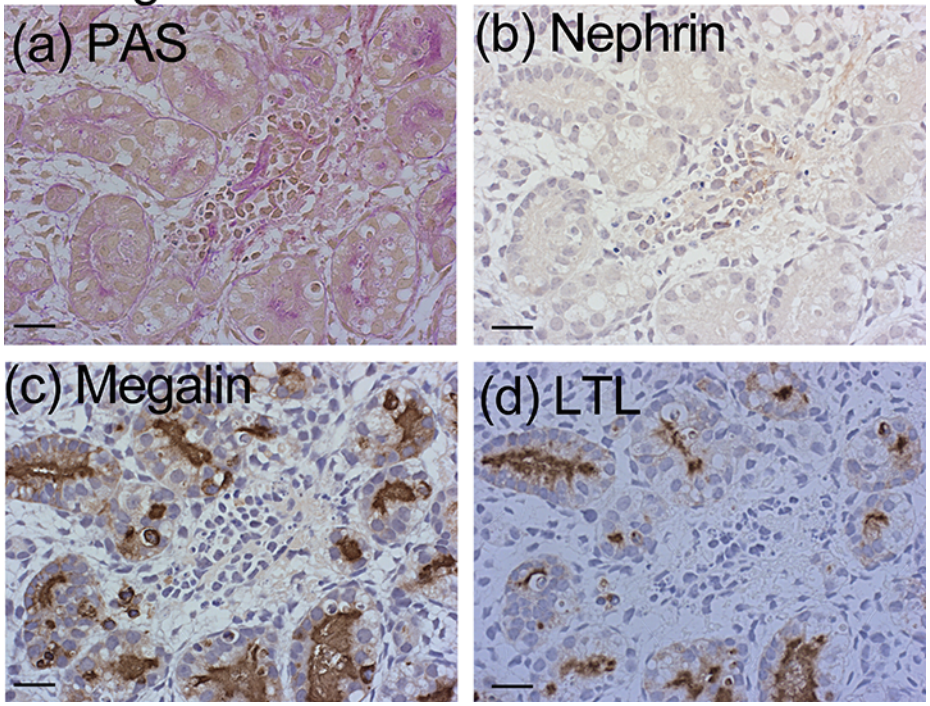

S1 Figure. Tubular markers in NEP25/Ribotag organoids. NEP25/Ribotag organoids were treated with LMB2 for 3 days. The expression level of nephrin is reduced, whereas megalin and LTL staining is retained. Panels (a–d) represent the equivalent visual fields of the serial sections.

Scale bar: 50 μm.
