# Supplementary material for: Podocyte injury damages podocytes in chimeric organoids
Source: PLoS One. 2026 Aug 31;21(8):e0337677. doi: 10.1371/journal.pone.0337677 (PMC13528954; doi:10.1371/journal.pone.0337677)
Supplement: Supplementary file 2 [file pone.0337677.s003.pdf]

S2 Figure

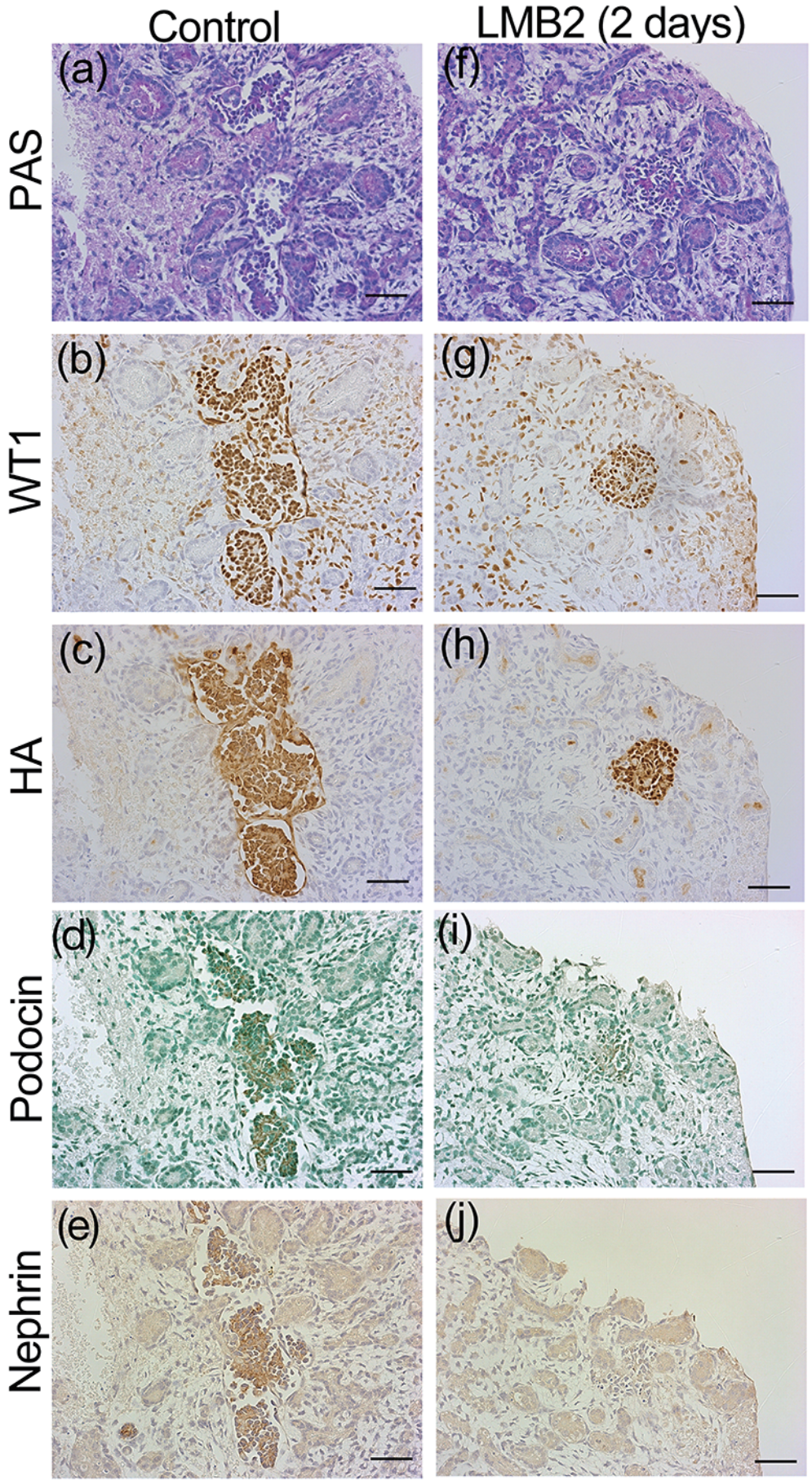

Figure S2. NEP25/Ribotag organoids treated with LMB2 treatment for 2 days.

In control organoids without LMB2, podocytes strongly express WT1, HA, podocin, and nephrin. After LMB2 treatment, a few podocyte clusters remained. They retained WT1 and HA expression, but the expression levels of podocin and nephrin were reduced. Panels (a–e) and (f–j) represent the equivalent visual fields of the serial sections. Quantification of podocin staining intensity is provided in Table S2D in S2 Table. Scale bar: 20 μm.
