# Supplementary material for: Podocyte injury damages podocytes in chimeric organoids
Source: PLoS One. 2026 Aug 31;21(8):e0337677. doi: 10.1371/journal.pone.0337677 (PMC13528954; doi:10.1371/journal.pone.0337677)
Supplement: Supplementary file 3 [file pone.0337677.s004.pdf]

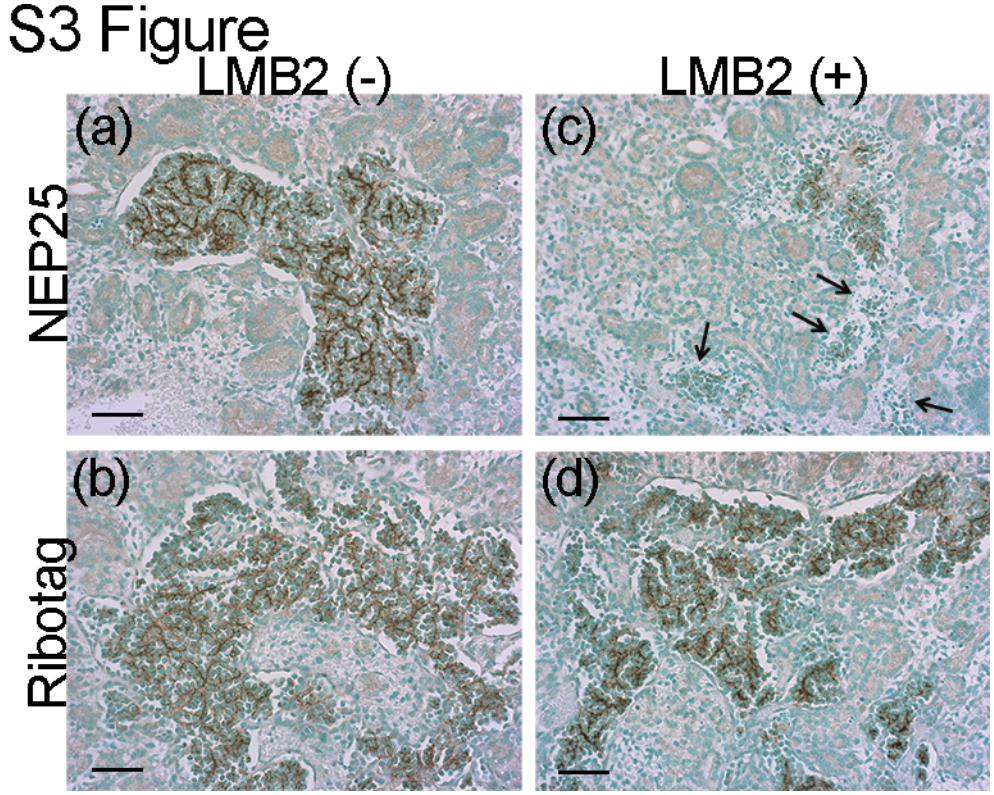

S3 Figure. Podocyte injury does not propagate between organoids.

NEP25 and Ribotag organoids were cocultured on a single Transwell membrane. Without LMB2, both organoid types express podocin (a, b). After 3 days of LMB2 treatment, the expression of podocin is markedly suppressed in NEP25 organoids (arrows, c) but not in Ribotag organoids (d). Quantification of podocin staining intensity is provided in Table S2E in S2 Table. Scale bar: 20 μm.
