# Supplementary material for: Podocyte injury damages podocytes in chimeric organoids
Source: PLoS One. 2026 Aug 31;21(8):e0337677. doi: 10.1371/journal.pone.0337677 (PMC13528954; doi:10.1371/journal.pone.0337677)
Supplement: Supplementary file 4 [file pone.0337677.s005.pdf]

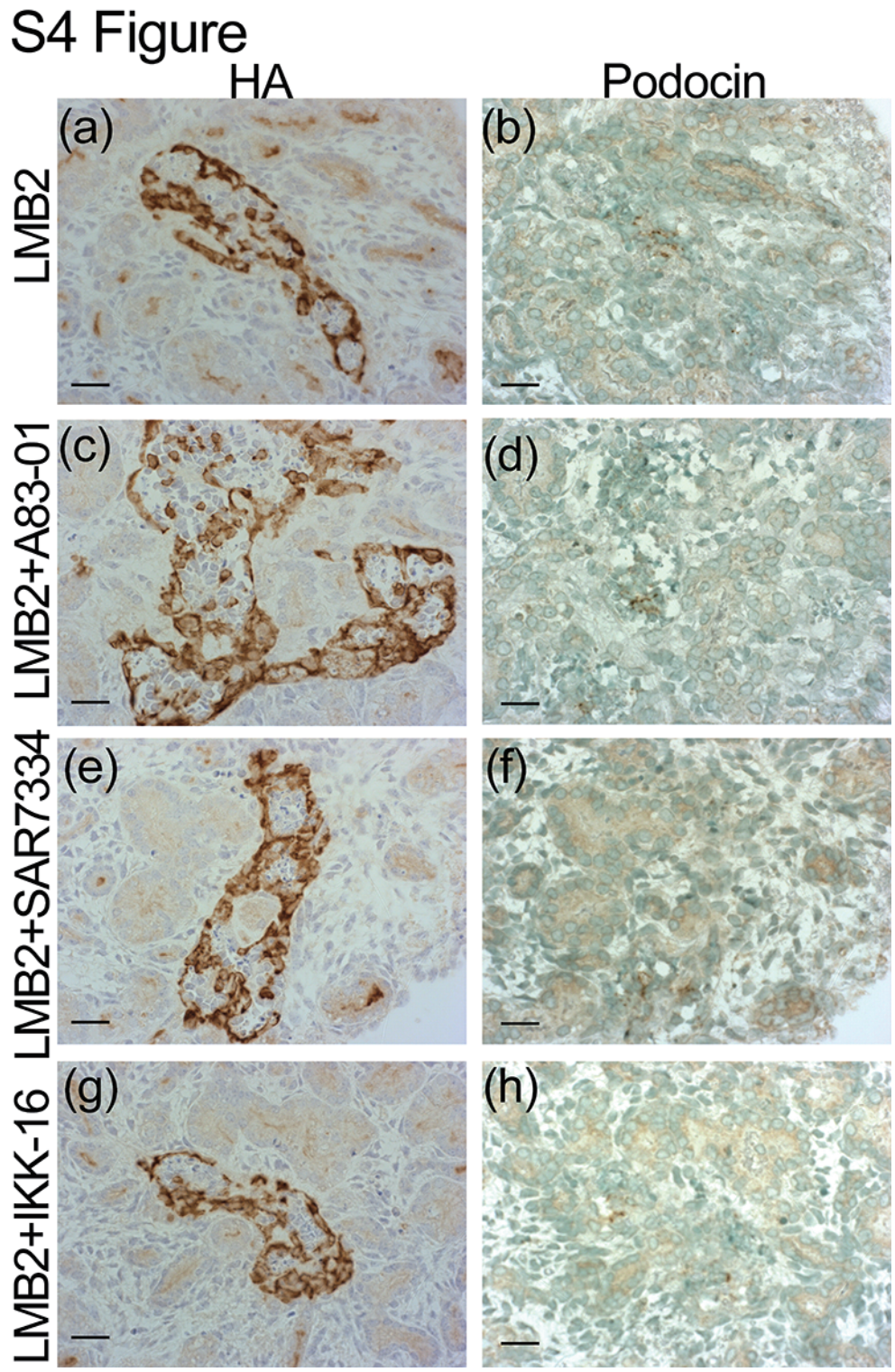

S4 Figure. Effects of inhibitors on indirect podocin suppression in chimeric organoids.

LMB2 treatment for 2 days reduces podocin staining (b) despite the presence of HA(+) podocytes (a). Cotreatment with A83-01 (TGF-β inhibitor) (c and d), SAR7334 (TRPC6 inhibitor)(e and f), or IKK-16 (NF-κB inhibitor)(g and h) does not restore podocin expression. Quantification of podocin staining intensity is provided in Table S2F in S2 Table. Scale bar: 50 μm.
