# Supplementary material for: Podocyte injury damages podocytes in chimeric organoids
Source: PLoS One. 2026 Aug 31;21(8):e0337677. doi: 10.1371/journal.pone.0337677 (PMC13528954; doi:10.1371/journal.pone.0337677)
Supplement: Supplementary file 5 [file pone.0337677.s006.pdf]

S5 Figure

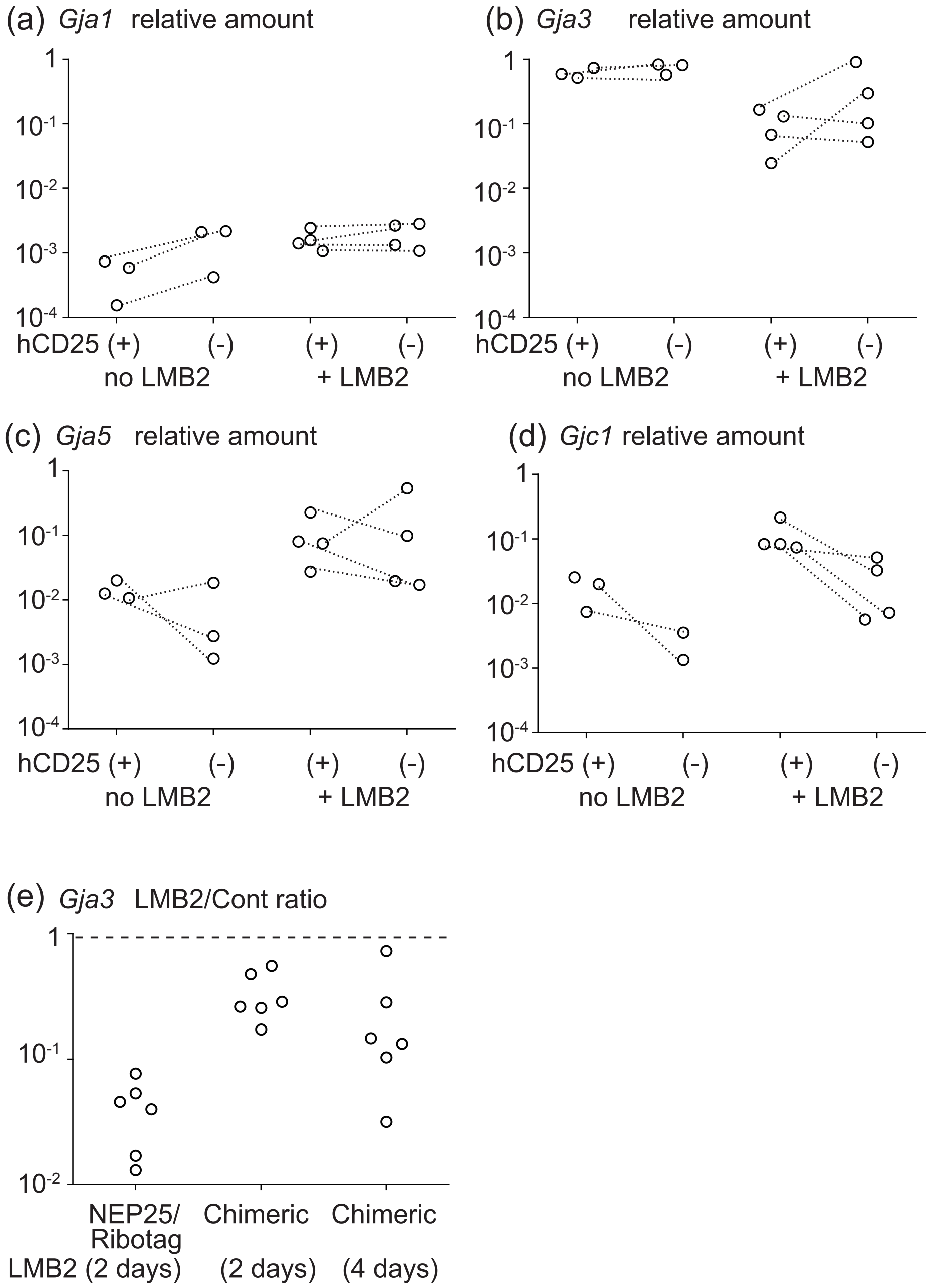

S5 Figure. Connexin mRNA expression in mosaic mice and organoids.

(a–d) hCD25(+) and hCD25(–) podocytes were isolated from mosaic mice either untreated (n = 3) or 7 days post-LMB2 injection (n = 5). Quantitative RT-PCR was performed using Actb as a control. Data are presented relative to heart RNA from a wild-type mouse. (e) Ratio of LMB2-treated to control polysomal RNA in NEP25/Ribotag organoids (n = 6) and chimeric organoids after 2 days (n = 6) or 4 days (n = 8) of LMB2 treatment. In NEP25/Ribotag organoids, IP RNA is derived from hCD25(+) podocytes. In chimeric organoids, IP RNA is derived from hCD25(−) podocytes. Gja3 mRNA was undetectable in two IP samples of chimeric organoids treated with LMB2 for 4 days, which are not shown in the graph. LMB2 significantly reduces the mRNA expression level of Gja3 in NEP25/Ribotag and chimeric organoids ($p < 0.01$).
