# Supplementary material for: Podocyte injury damages podocytes in chimeric organoids
Source: PLoS One. 2026 Aug 31;21(8):e0337677. doi: 10.1371/journal.pone.0337677 (PMC13528954; doi:10.1371/journal.pone.0337677)
Supplement: Supplementary file 6 [file pone.0337677.s007.pdf]

S6 Figure

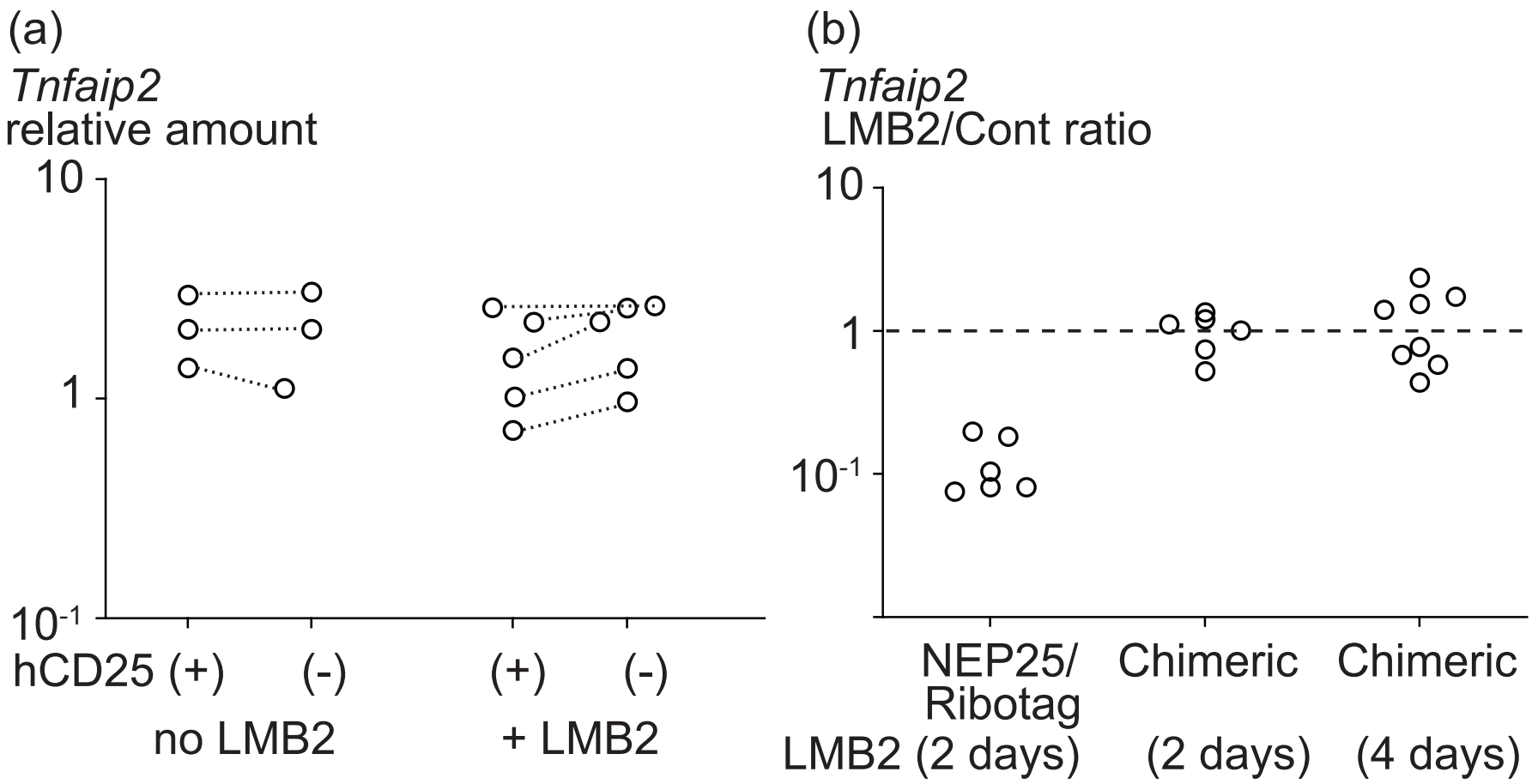

S6 Figure. Tnfaip2 mRNA expression in mosaic mice and organoids. (a) hCD25(+) and hCD25(−) podocytes were isolated from mosaic mice without LMB2 (n = 3) or 7 days post-LMB2 injection (n = 5). Tnfaip2 mRNA levels were measured via quantitative RT-PCR, normalized to Actb, and expressed relative to total kidney RNA from a wild-type mouse. (b) Ratio of LMB2-treated to control polysomal RNA in NEP25/Ribotag organoids (n = 6) and chimeric organoids after 2 days (n = 6) or 4 days (n = 8) of LMB2 treatment. In NEP25/Ribotag organoids, IP RNA is derived from hCD25(+) podocytes. In chimeric organoids, IP RNA is derived from hCD25(−) podocytes. LMB2 significantly reduces the mRNA expression level of Tnfaip2 in NEP25/Ribotag organoids ($p < 0.01$) but not in chimeric organoids.
