# Supplementary material for: Podocyte injury damages podocytes in chimeric organoids
Source: PLoS One. 2026 Aug 31;21(8):e0337677. doi: 10.1371/journal.pone.0337677 (PMC13528954; doi:10.1371/journal.pone.0337677)
Supplement: Supplementary file 7 [file pone.0337677.s008.pdf]

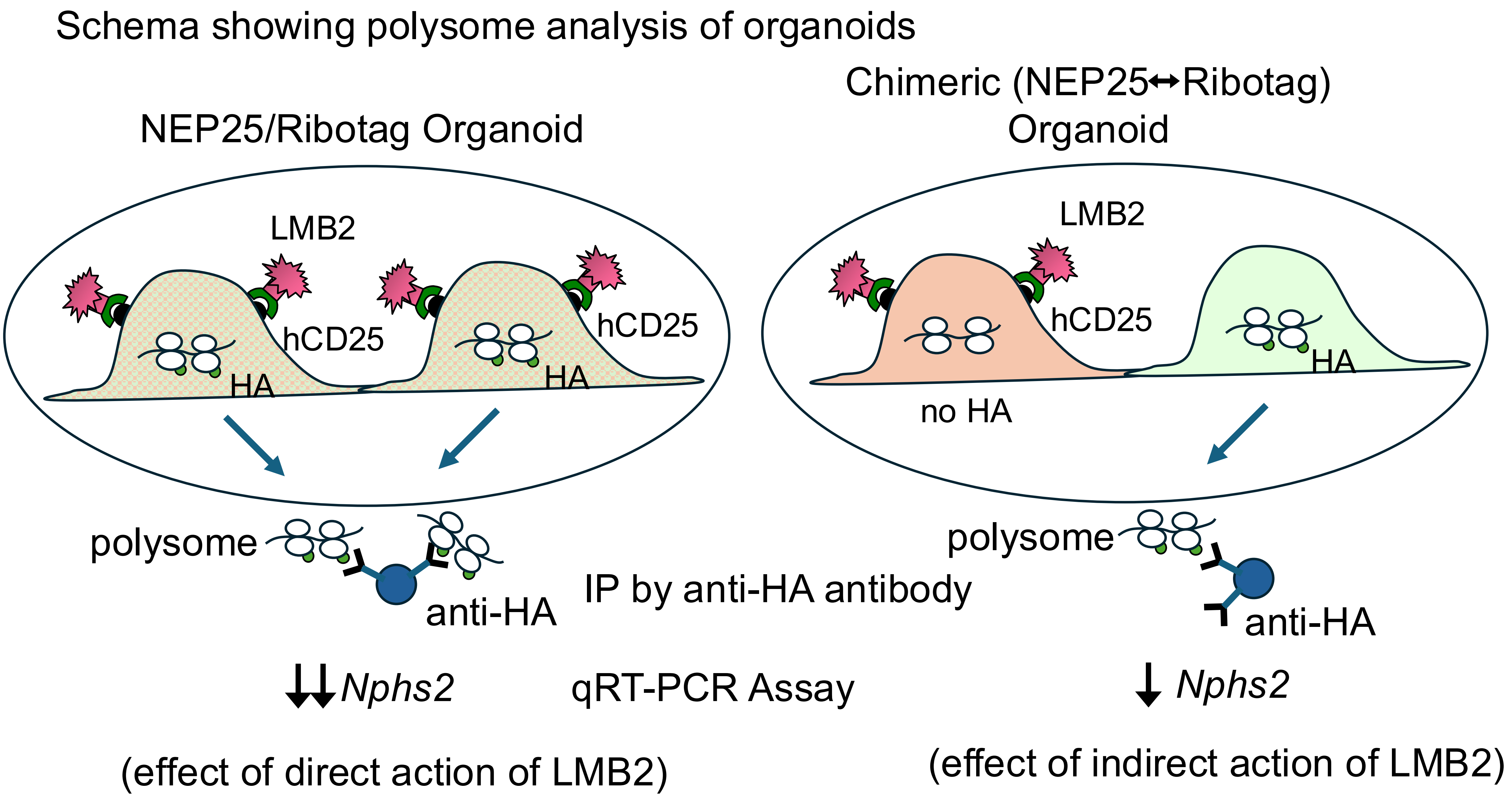
Schema showing polysome analysis of organoids
NEP25/Ribotag Organoid
Chimeric (NEP25↔Ribotag)
Organoid
LMB2
hCD25
HA
hCD25
HA
LMB2
hCD25
HA
no HA
polysome
anti-HA
IP by anti-HA antibody
polysome
anti-HA
↓↓Nphs2
qRT-PCR Assay
↓ Nphs2
(effect of direct action of LMB2)
(effect of indirect action of LMB2)
