# Supplementary material for: Podocyte injury damages podocytes in chimeric organoids
Source: PLoS One. 2026 Aug 31;21(8):e0337677. doi: 10.1371/journal.pone.0337677 (PMC13528954; doi:10.1371/journal.pone.0337677)
Supplement: Supplementary file 8 [file pone.0337677.s001.docx]

| Transgene | primer 1 | primer 2 | product size |
| --- | --- | --- | --- |
| Nphs1-hCD25 | GTTTATTATCAGTGCGTCCAG | CTTGTCATCGTCGTCCTTGTA | 409 bp |
| Nphs1-Cre | AGGTTCGTTCACTCATGGA | TCGACCAGTTTAGTTACCC | 235 bp |
| Rpl22^tm1.1Psam^ | GGGAGGCTTGCTGGATATG | TTTCCAGACACAGGCTAAGTACAC | 290 bp (243 bp in wild-type) |

S1 Table

Supplementary Tables A-D

Table A

PCR primers for genotyping

Table B

Antibodies

|  | Company | Catalogue | dilution, concentration | pretreatment |
| --- | --- | --- | --- | --- |
| for paraffin sections | |  |  |  |
| Nephrin | Progen | GP-N2 | 1/100 | heat |
| HA-tag | Cell Signaling technology | #3724 | 1/8000 | heat |
| hCD25 | Thermo Scientific | MS-1088 | (1/20) | heat |
| WT1 | Abcam | ab89901 | 1/2000 | heat |
| Podocalyxin | R&D systems | MAB1556 | 1/8000 | trypsin |
| podocin | Sigma-Aldrich | P0372 | 1/5000 | pepsin |
| Biotin-LTL | Vector | B-1325 | 10 𝝻g/ml |  |
|  |  |  |  |  |
| for fluorescent staining | |  |  |  |
| FITC-LTL | Vector | FL-1321 | 10 𝝻g/ml |  |
| WT1(f) | Abcam | ab89901 | 1/2000 |  |
|  |  |  |  |  |
| for immunoprecipitation | |  |  |  |
| HA.11 epitope | BioLegend | 901501 | 12.5 𝝻g/ml |  |

Table C

Primers for quantitative PCR

| gene | Primer1 | Primer2 |
| --- | --- | --- |
| Actb | ATGAGCTGCCTGACGGCCAGGTCATC | TGGTACCACCAGACAGCACTGTGTTG |
| Gadd45b | CTGCCTCCTGGTCACGAA | TTGCCTCTGCTCTCTTCACA |
| P2rx7 | AGCAGAGGTGACGGAGAATG | CTGCACTTGGCCTTCTGACT |
| Cxcl1 | GACTCCAGCCACACTCCAAC | TGACAGCGCAGCTCATTG |
| Egr1 | CCTATGAGCACCTGACCACA | TCGTTTGGCTGGGATAACTC |
| Lrp2 | AGGCTCCGACTTCGTAACTG | GCATGGTGATGGGGAGTT |
| Tnfaip2 | GCCGAAAGCTACCACTACCA | GAAGCTCCTGTGCCAACTTC |
| Gja1 | GTGCCGGCTTCACTTTCA | GGAGTAGGCTTGGACCTTGTC |
| Gja3 | CCCCAGTGTCTGAGATCGAG | AGGCCGAACAAGAGCAAG |
| Gja5 | CAGGGAGGAGGAAAGGAAGC | ACTGTGGAGTGCTTGTGGAC |
| Gjc1 | ACAGGAGTTCTGGTGAACAGG | TCTCCTCTAGCAGGCGAGTC |

Table D

TaqMan Gene Expression Assays

| gene | Company | Assay ID |
| --- | --- | --- |
| Nphs1 | ThermoFisher | Mm00497828_m1 |
| Nphs2 | ThermoFisher | Mm00499929_m1 |
| Wt1 | ThermoFisher | Mm00460570_m1 |
| hCD25 | ThermoFisher | Hs00166229_m1 |
