# Supplementary material for: Podocyte injury damages podocytes in chimeric organoids
Source: PLoS One. 2026 Aug 31;21(8):e0337677. doi: 10.1371/journal.pone.0337677 (PMC13528954; doi:10.1371/journal.pone.0337677)
Supplement: Supplementary file 9 [file pone.0337677.s011.docx]

**Reviewer 1**

| **Reviewer Recommendation Term:** | Major Revision |
| --- | --- |
| **Rate Review:** | 0 |
| **Custom Review Question(s):** \| **Response**; **Comments to the Author** 1. Is the manuscript technically sound, and do the data support the conclusions? \| Yes; 2. Has the statistical analysis been performed appropriately and rigorously? \| Yes; 3. Have the authors made all data underlying the findings in their manuscript fully available? \| Yes; 4. Is the manuscript presented in an intelligible fashion and written in standard English? \| Yes; 5. Review Comments to the Author \| The manuscript addresses an important question regarding the mechanisms by which podocyte injury propagates locally within the glomerulus. By generating chimeric kidney organoids composed of LMB2-sensitive and -resistant podocytes, the authors successfully develop an in vitro system to explore podocyte–podocyte injury transmission independent of filtration forces and other glomerular cell types. Overall, the experimental approach is technically sound, and the data support the major conclusion that direct cellular proximity is required for secondary podocyte injury Points that require clarification or improvement: Quantification of imaging data: Many key observations—particularly loss of podocin or WT1 staining in indirectly injured podocytes—are presented solely as representative images. Quantitative morphometric data (e.g., podocyte counts, signal intensity, or area of staining) would significantly strengthen the conclusions. Incomplete reporting of sample sizes in imaging experiments: While qRT-PCR analyses include clear biological replicates, the number of organoids examined for each imaging result is not specified. This information is necessary to evaluate reproducibility. Data availability requirements: The manuscript states that all data are provided within the article and supporting files, but underlying raw data (e.g., Ct values for qPCR, numerical replicate values, or quantifications of immunostaining) are not included. For full compliance with PLOS data policies, these datasets should be provided as supplementary files. Statistical analysis considerations: The statistical methods for polysome qRT-PCR experiments are appropriate, but assumptions (e.g., normality) are not discussed. In addition, negative inhibitor experiments (gap junction inhibition, TRPC6, TGF-β, NF-κB inhibition) are evaluated qualitatively; quantitative assessment would make the interpretation more robust. Clarity and style of writing: The manuscript is generally well written; however, several sentences are overly long or densely structured. Minor grammatical and stylistic inconsistencies are present throughout the text, and the figure legends could be clarified further. These issues do not obscure the scientific message but should be addressed during revision. Mechanistic implications: While the authors appropriately acknowledge that the precise signaling mechanism remains unresolved, the Discussion could benefit from a more explicit statement that the current data rule out some pathways (e.g., GJ blockade) only partially, as the inhibitor experiments do not represent a comprehensive mechanistic screen. Overall assessment: The manuscript presents novel and valuable findings demonstrating that podocyte injury can propagate between directly adjacent podocytes in organoids, independently of filtration pressure or non-podocyte glomerular cells. The work is conceptually significant and suitable for publication in PLOS ONE after the concerns outlined above are addressed. The most important improvements needed relate to data quantification, data availability, and clarification of methods and writing.; 6. PLOS authors have the option to publish the peer review history of their article ([what does this mean?](https://journals.plos.org/plosone/s/editorial-and-peer-review-process#loc-peer-review-history)). If published, this will include your full peer review and any attached files. \| No; **Confidential to Editor** 1. Do you have any potential or perceived competing interests that may influence your review? \| I have no competing interests.; 2. Did you receive any assistance in preparing this review (e.g. from a post-doc or graduate student)? If yes, please include their name below. \| No, I did not receive any assistance.; 3. If accepted, do you think this submission should be highlighted on the PLOS ONE website? PLOS ONE does not evaluate manuscripts based on perceived significance or readership. We aim to provide tools for readers to filter and evaluate our publications. (optional) \| Yes, on a more specific subject area page (e.g. Biochemistry, Atmospheric Science); **Do you want to get recognition for this review on** [**a Web of Science researcher profile**](https://clarivate.com/products/scientific-and-academic-research/research-discovery-and-workflow-solutions/researcher-profiles/)**?** \| Yes | |

Reviewer 2

| **Reviewer Recommendation Term:** | Minor Revision |
| --- | --- |
| **Rate Review:** | 0 |
| **Custom Review Question(s):** \| **Response**; **Comments to the Author** 1. Is the manuscript technically sound, and do the data support the conclusions? \| Yes; 2. Has the statistical analysis been performed appropriately and rigorously? \| I Don't Know; 3. Have the authors made all data underlying the findings in their manuscript fully available? \| Yes; 4. Is the manuscript presented in an intelligible fashion and written in standard English? \| Yes; 5. Review Comments to the Author \| The topic sounds good. The hypothesis put forward is highly relevant. The methods used are well designed. The results are well expressed and discussed. Here are my specific comments: * introduction - the stage for the study is well-set by highlighting the importance of podocyte injury in glomerulosclerosis - the authors provide a concise overview of previous studies including their previous work which helps understanding the context of this study - please complete with the insertion of the corresponding references (ex: last sentence for the first paragraph et the second paragraph) * methods - methods are well-written - please provide references for the adopted methods - please give more details about somes methods such as the immunoprecipitation procedure and the use of the RNeasy Micro Kit. - Can the authors present a graphical summary of the methods used? This will be very helpful in facilitating understanding for the reader. * results - results are well-presented in a clear and concise manner - please identify the statistically different groups in the figures.; 6. PLOS authors have the option to publish the peer review history of their article ([what does this mean?](https://journals.plos.org/plosone/s/editorial-and-peer-review-process#loc-peer-review-history)). If published, this will include your full peer review and any attached files. \| No; **Confidential to Editor** 1. Do you have any potential or perceived competing interests that may influence your review? \| The topic sounds good. The hypothesis put forward is highly relevant. The methods used are well designed. The results are well expressed and discussed. Here are my specific comments: * introduction - the stage for the study is well-set by highlighting the importance of podocyte injury in glomerulosclerosis - the authors provide a concise overview of previous studies including their previous work which helps understanding the context of this study - please complete with the insertion of the corresponding references (ex: last sentence for the first paragraph et the second paragraph) * methods - methods are well-written - please provide references for the adopted methods - please give more details about somes methods such as the immunoprecipitation procedure and the use of the RNeasy Micro Kit. - Can the authors present a graphical summary of the methods used? This will be very helpful in facilitating understanding for the reader. * results - results are well-presented in a clear and concise manner - please identify the statistically different groups in the figures.; 2. Did you receive any assistance in preparing this review (e.g. from a post-doc or graduate student)? If yes, please include their name below. \| None; 3. If accepted, do you think this submission should be highlighted on the PLOS ONE website? PLOS ONE does not evaluate manuscripts based on perceived significance or readership. We aim to provide tools for readers to filter and evaluate our publications. (optional) \| Yes, on a broad subject area page (e.g. Biology, Earth Sciences) | |
