# Supplementary material for: Podocyte injury damages podocytes in chimeric organoids
Source: PLoS One. 2026 Aug 31;21(8):e0337677. doi: 10.1371/journal.pone.0337677 (PMC13528954; doi:10.1371/journal.pone.0337677)
Supplement: Supplementary file 10 [file pone.0337677.s013.docx]

**Reviewer 3**

| **Reviewer Recommendation Term:** | Minor Revision |
| --- | --- |
| **Rate Review:** | 0 |
| **Custom Review Question(s):** \| **Response**; **Comments to the Author** 1. If the authors have adequately addressed your comments raised in a previous round of review and you feel that this manuscript is now acceptable for publication, you may indicate that here to bypass the “Comments to the Author” section, enter your conflict of interest statement in the “Confidential to Editor” section, and submit your "Accept" recommendation. \|; 2. Is the manuscript technically sound, and do the data support the conclusions? \|; 3. Has the statistical analysis been performed appropriately and rigorously? \|; 4. Have the authors made all data underlying the findings in their manuscript fully available? \|; 5. Is the manuscript presented in an intelligible fashion and written in standard English? \|; 6. Review Comments to the Author \| The manuscript presents a well-designed experimental study investigating the propagation of podocyte injury using chimeric kidney organoids. The use of a mosaic organoid system to isolate direct cell–cell interactions is a notable strength, and the findings provide meaningful insight into mechanisms underlying local injury spread in kidney disease. The combination of imaging, molecular analysis, and functional assays supports the conclusions. Overall, the study is of good quality, and only minor revisions are required to further improve clarity and presentation. 1. Some sentences, particularly in the introduction and discussion sections, are slightly long and could be simplified to improve readability. 2. The distinction between “indirect injury,” “secondary injury,” and “bystander effect” could be clarified and used more consistently throughout the manuscript. 3. The novelty of the study could be stated more concisely, especially in relation to previous in vivo findings mentioned in the introduction. 4. The description of the organoid model and its limitations (e.g., absence of endothelial and mesangial cells) could be discussed a bit more clearly. 5. Figure captions (e.g., Figs. 2 and 5) could be slightly expanded to better guide the reader in interpreting key findings such as the loss of podocin in non-targeted cells. 6. The results section would benefit from a bit more explanation of the biological reasoning behind the observed trends. 7. The discussion of possible mechanisms (e.g., gap junctions, TNTs) could be presented more cautiously as potential explanations rather than definitive conclusions. 8. Minor typographical and formatting inconsistencies (e.g., spacing, notation, gene names) should be carefully revised.; 7. **Do you want your identity to be public for this peer review?** For information about this choice, including consent withdrawal, please see our [Privacy Policy](https://www.plos.org/privacy-policy). \| No; **Confidential to Editor** 1. Do you have any potential or perceived competing interests that may influence your review? \| I have no competing interests.; 2. Did you receive any assistance in preparing this review (e.g. from a post-doc or graduate student)? \|; 3. If accepted, do you think this submission should be highlighted on the PLOS ONE website? \|; **Do you want to get recognition for this review on** [**a Web of Science researcher profile**](https://clarivate.com/products/scientific-and-academic-research/research-discovery-and-workflow-solutions/researcher-profiles/)**?** \| Yes | |
