# Supplementary material for: Podocyte injury damages podocytes in chimeric organoids
Source: PLoS One. 2026 Aug 31;21(8):e0337677. doi: 10.1371/journal.pone.0337677 (PMC13528954; doi:10.1371/journal.pone.0337677)
Supplement: Supplementary file 11 [file pone.0337677.s014.docx]

**Response to Reviewers**

**Manuscript Number:** PONE-D-25-60672

**Title:** Podocyte injury damages podocytes in chimeric organoids

**Corresponding Author:** Taiji Matsusaka

Dear Dr. Silambanan and Editors of PLOS ONE,

We would like to thank the Editor and the Reviewers for their positive and encouraging evaluation of our manuscript. We appreciate the insightful and constructive comments provided, which have significantly helped us improve the quality and clarity of our work.

In response to the reviewers’ suggestions, we have performed additional experiments, including podocin and HA immunostaining, re-evaluated our statistical analyses, and provided raw data for transparency. We have also added a new supplementary figure illustrating our methodology and included additional references. The manuscript has been extensively edited for clarity, flow, and grammatical consistency.

Our point-by-point responses to the specific concerns raised by the reviewers are detailed below.

**Response to Reviewer 1**

**1. Quantification of imaging data and sample sizes:**

*Many key observations—particularly loss of podocin or WT1 staining—are presented solely as representative images. Quantitative morphometric data and reporting of sample sizes are necessary.*

**Response:** We appreciate the reviewer’s constructive suggestion. To enhance the robustness of our findings, we have now performed a quantitative analysis of podocin signal intensity for **Figures 2, 5, 6, S2, S3, and S4**.

To ensure accurate quantification, we addressed two technical challenges: 1) podocin intensity varies with the area of podocyte clusters within a given section, and 2) the baseline differentiation of organoids can vary between batches. To correct for these factors, we normalized the podocin-positive intensity to the HA-positive area in adjacent sections. The HA-tag, fused to a mitochondrial protein specifically in podocytes, serves as a stable internal marker because its expression is more resistant to LMB2-induced injury compared to podocyte differentiation markers. For **S3 Figure**, podocin intensity was normalized to the total organoid area, as NEP25 organoids lack the HA tag.

The quantitative data for each individual organoid are detailed in S2 Table, which allows for a clear assessment of the sample size for each experimental group. Typically, we analyzed n = 2-4 organoids per condition to ensure reproducibility. While the sample size for some groups was constrained by the complexity of the organoid assays, the quantitative results consistently demonstrate that podocin expression is significantly suppressed by LMB2 in both NEP25/RiboTag and chimeric organoids. Furthermore, this suppression was not attenuated by CBX or other tested inhibitors, supporting our qualitative observations. We have updated the Methods section and Figure Legends to reflect these quantitative analyses.

**2. Data availability requirements:**

*Raw data (e.g., Ct values for qPCR, numerical replicate values, or quantifications of immunostaining) should be provided.*

**Response:** In compliance with PLOS ONE’s data policy, we have provided all raw data. **S3 Table** now includes Ct values for all qPCR experiments, and **S2 Table** contains numerical replicate values and quantifications for all podocin immunostaining.

**3. Statistical analysis considerations:**

*Assumptions (e.g., normality) for polysome qRT-PCR experiments are not discussed.*

**Response:** We thank the reviewer for this important point. Since the original qPCR data exhibited a right-skewed distribution, we performed logarithmic transformations to stabilize the variance and approximate a normal distribution.

In the revised **Fig. 3**, we now present scatter plots with geometric means. While log-transformation was generally effective, we evaluated the normality of residuals and noted that for certain genes (e.g., *Nphs1* and *Wt1* in Fig. 3d), the residuals remained slightly skewed. However, alternative transformations (square-root or reciprocal) provided a marginally better fit to the normal distribution as shown below.However, the statistical significance remained unchanged regardless of the transformation method used. For instance, the adjusted P-values for Nphs1, Nphs2, Wt1, and Gadd45b remained consistently below 0.05 (see the table below). This confirms the robustness of our conclusions. We have added a statement regarding these transformations to the Figure 3 legend.

**4. Clarity and style of writing:**

*Several sentences are overly long or densely structured. Minor grammatical and stylistic inconsistencies are present.*

**Response:** We have conducted a comprehensive revision of the manuscript to improve readability. Long sentences have been shortened, and we have refined technical descriptions to be more concise. All Figure Legends have also been revised for clarity regarding experimental groups and statistical analyses.

**5. Mechanistic implications:**

*The Discussion could benefit from a more explicit statement that the current data rule out some pathways only partially.*

**Response:** We agree with the reviewer’s insightful point. While our inhibitor experiments (including gap junction blockade) did not show a rescue effect, we acknowledge that this does not constitute an exhaustive mechanistic screen. We have added a statement to the **Discussion** clarifying that these pathways are only partially ruled out and acknowledging this as a limitation of the study.

**Response to Reviewer 2**

**1. References in Introduction:**

*The corresponding references should be inserted after the last sentence for the first and second paragraphs.*

**Response:** We have now included the relevant references at the end of the first and second paragraphs of the Introduction as requested.

**2. Methods details and graphical summary:**

*Provide more details about IP and RNeasy Micro Kit. Can the authors present a graphical summary of the methods?*

**Response:** We have expanded the Methods section to include more detail on the immunoprecipitation procedure and the use of the RNeasy Micro Kit, including additional citations. Furthermore, we have added **S7 Figure**, which provides a schematic overview of the polysome analysis in chimeric organoids to facilitate reader understanding.

**3. Results - Statistical identification:**

*Please identify the statistically different groups in the figures.*

**Response:** We have updated **Figure 3** to clearly indicate statistically significant groups using asterisks (*).

We believe that these revisions have strengthened the manuscript and addressed all the concerns raised by the reviewers. We hope the revised version is now suitable for publication in *PLOS ONE*.

Sincerely,

**Taiji Matsusaka, MD, PhD**

Department of Physiology

Tokai University School of Medicine
