# Supplementary material for: Podocyte injury damages podocytes in chimeric organoids
Source: PLoS One. 2026 Aug 31;21(8):e0337677. doi: 10.1371/journal.pone.0337677 (PMC13528954; doi:10.1371/journal.pone.0337677)
Supplement: Supplementary file 12 [file pone.0337677.s015.docx]

**May 28, 2026**

Dear Editor and Reviewers,

We would like to express our sincere gratitude to the reviewers for their constructive comments and highly positive evaluation of our manuscript entitled "Podocyte injury damages podocytes in chimeric organoids" (Manuscript ID: PONE-D-25-60672R).

We have carefully addressed all the points raised by Reviewer #3. The manuscript has been revised accordingly, and we believe these changes have significantly improved the clarity and quality of our manuscript.

**Response to Academic Editor**

**Journal Requirement / Recommendation:** *If applicable, we recommend that you deposit your laboratory protocols in protocols.io to enhance the reproducibility of your results. Protocols.io assigns your protocol its own identifier (DOI) so that it can be cited independently in the future.*

**Response:** We sincerely appreciate the editor’s valuable recommendation to utilize protocols.io to enhance reproducibility. Regarding the protocol for generating the kidney organoids, which is a key characteristic of our study, we would like to clarify that the detailed step-by-step procedures have already been fully established and previously published by our co-authors (referred to as Ref. 18 in the manuscript). Because this robust protocol is already available to the public and properly cited in our Methods section, we have not deposited it as a new protocol on protocols.io. We hope the editor finds this explanation acceptable, as the reproducibility of the method is already well-secured through our previous publication.

Below is our point-by-point response to the reviewer’s comments.

**Response to Reviewer #3**

**General Comment:**

*The manuscript presents a well-designed experimental study investigating the propagation of podocyte injury using chimeric kidney organoids. The use of a mosaic organoid system to isolate direct cell–cell interactions is a notable strength, and the findings provide meaningful insight into mechanisms underlying local injury spread in kidney disease. The combination of imaging, molecular analysis, and functional assays supports the conclusions. Overall, the study is of good quality, and only minor revisions are required to further improve clarity and presentation.*

**Response:** We sincerely thank the reviewer for the highly encouraging feedback and appreciation of our mosaic organoid system. We agree that the suggested minor revisions greatly enhance the clarity and presentation of our findings.

**Comment 1:** *Some sentences, particularly in the introduction and discussion sections, are slightly long and could be simplified to improve readability.*

**Response:** We agree with the reviewer's assessment. We have simplified several long and complex sentences throughout the Introduction and Discussion sections to enhance readability.

- **In the Introduction [Lines 50-52]:** We split the sentence regarding LMB2 clearance into two separate sentences:
  - *Old:* "Notably, a single injection of LMB2 results in progressive kidney injury over several weeks, even though the toxin is rapidly cleared and podocytes with activated caspase are eliminated within seven days."
  - *Revised:* "Notably, a single injection of LMB2 results in progressive kidney injury extending over several weeks. This progression occurs despite the rapid clearance of the toxin and the elimination of caspase-activated podocytes within seven days."
- **In the Discussion [Lines 280-282]:** We similarly simplified the sentence describing injury propagation:
  - *Old:* "Our findings indicate that podocytes directly injured by LMB2 can induce secondary damage to neighboring, initially intact podocytes within chimeric organoids, despite the absence of glomerular filtration or other glomerular cell types."
  - *Revised:* "Our findings indicate that podocytes directly injured by LMB2 induce indirect damage to neighboring, initially intact podocytes within chimeric organoids. Importantly, this propagation occurs even in the absence of glomerular filtration or other glomerular cell types."

**Comment 2:** *The distinction between “indirect injury,” “secondary injury,” and “bystander effect” could be clarified and used more consistently throughout the manuscript.*

**Response:** Thank you for this excellent suggestion. To ensure conceptual consistency, we have standardized our terminology by shifting our preference to the term **"indirect injury"** throughout the entire manuscript, including the Abstract, Results, Discussion, and Figure Legends.

**Comment 3:** *The novelty of the study could be stated more concisely, especially in relation to previous in vivo findings mentioned in the introduction.*

**Response:** We have revised the summary of our findings at the end of the Introduction to state the novelty of this study more concisely and clearly. We emphasized that our organoid model successfully isolates direct cell-cell interactions from confounding *in vivo* environmental factors.

- *Revised text [Lines 74-77]:* "Using this new *in vitro* system, we successfully isolated direct podocyte-to-podocyte communication from confounding *in vivo* factors, such as filtration pressure and other types of cells. We demonstrate for the first time *in vitro* that podocyte injury propagation is an autonomous, self-sustaining process driven by direct podocyte-to-podocyte interactions."

**Comment 4:** *The description of the organoid model and its limitations (e.g., absence of endothelial and mesangial cells) could be discussed a bit more clearly.*

**Response:** As suggested, we have expanded the "Limitations" section in the Discussion to provide a more comprehensive and rigorous description of our organoid model. Specifically, we have explicitly stated that our organoids are derived exclusively from nephron progenitor cells (thereby lacking interstitial and ureteric bud derivatives) and have discussed how the absence of endothelial and mesangial cells represents a simplified but advantageous system that still warrants cautious interpretation regarding native diseases.

- *Revised text [Lines 274-279]:* "Third, our organoids are generated exclusively from nephron progenitor cells, thereby lacking components derived from interstitial progenitors and ureteric buds. Consequently, the tissue architecture of these organoids differs from that of the *in vivo* counterpart. While the absence of endothelial and mesangial cells allowed us to isolate and prove that they are not required for injury propagation, in native glomerular diseases, these cell types likely modulate the autonomous podocyte pathways described here."

**Comment 5:** *Figure captions (e.g., Figs. 2 and 5) could be slightly expanded to better guide the reader in interpreting key findings such as the loss of podocin in non-targeted cells.*

**Response:** We have expanded the legends for Figures 2 and 5 to better guide the reader in interpreting the cell-type specificity and the visual evidence of indirect injury propagation.

- *Revised Fig. 2 Legend [Lines 420-421]:* "...LMB2 treatment causes the loss of cluster morphology and podocyte marker expression, **whereas proximal tubule structures (LTL-positive) remain unaffected, demonstrating the cell-type specificity of the immunotoxin.**"
- *Revised Fig. 5 Legend [Lines 441-442]:* "Note the complete loss of hCD25(+) podocytes (d) and the indirect reduction of podocin staining (f) in the persisting HA(+) podocytes (e), **visually demonstrating the propagation of injury to non-targeted neighboring cells within the same glomerular-like structure.**"

**Comment 6:** *The results section would benefit from a bit more explanation of the biological reasoning behind the observed trends.*

**Response:** We agree with the reviewer's comment. To provide a clearer biological context for the observed gene expression trends, we have added a reference to previous *in vivo* findings, clarifying that the expression changes in our organoid model closely mirror those observed in chimeric mice. Additionally, we have explicitly stated the biological reasoning for targeting gap junctions in our inhibitor experiments.

The Results section has been revised as follows:

- In the Results section [Lines 202-203]: We added a clause to contextualize the gene expression changes with our previous *in vivo* data:
  - Old: "Following LMB2 treatment, these indirectly affected podocytes displayed reduced *Nphs1*, *Nphs2*, and *Wt1* expression levels and increased *Gadd45b* expression level."
  - Revised: "Following LMB2 treatment, these indirectly affected podocytes displayed reduced *Nphs1*, *Nphs2*, and *Wt1* expression levels and increased *Gadd45b* expression level, which is consistent with indirectly injured podocyte profiles in chimeric mice [13]."
- In the Results section [Lines 203-204]: We provided the biological rationale for examining gap junctions in the context of indirect injury propagation:
  - Added text: **Because it is frequently reported that gap junctions mediate bystander killing in other tissues by transferring cytotoxic molecules,** we hypothesized that intercellular signaling through gap junctions might mediate the indirect injury."

**Comment 7:** *The discussion of possible mechanisms (e.g., gap junctions, TNTs) could be presented more cautiously as potential explanations rather than definitive conclusions.*

**Response:** We agree with the reviewer and have softened our tone in the Discussion section to present gap junctions and tunneling nanotubes (TNTs) more cautiously as potential hypotheses rather than definitive conclusions.

- *Revised text for Gap Junctions [Lines 249-252]:* "**While these pharmacological results suggest** that gap junctions may not be the principal mediators of bystander injury in this model, **further genetic validation would be required to definitively exclude their contribution.**"
- *Revised text for TNTs [Lines 263-268]:* " Under such conditions**, it is conceivable that** injury signals may be transmitted from hCD25-positive to hCD25-negative podocytes via TNTs." …. from mosaic mice (S6 Figure). “**Direct assessment of TNT involvement remains an objective for future studies.”**

**Comment 8:** *Minor typographical and formatting inconsistencies (e.g., spacing, notation, gene names) should be carefully revised.*

**Response:** We have thoroughly reviewed the entire manuscript and corrected all typographical errors and formatting inconsistencies. Specifically, we have:

1. Corrected the gene name typo from *Npsh2* to *Nphs2* [Line 185].
2. Standardized the hyphenation for "2-day treatment" to match "4-day LMB2 treatment" [Line 186].
3. Unified the style for supplemental figure citations to (S2 Figure) throughout the text [Line 179].
4. Unified the style of unit (mL).
5. Corrected the spacing around the statistical symbols (e.g., *p*-value < 0.05) [Line 151].
6. Corrected spelling of “Schema” [Line 461].

**Additional Revision by the Authors:** While revising the manuscript, we noticed that the placement of the following sentences was inappropriate, given that most of the inhibitor experiments were performed using the 2-day LMB2 treatment protocol:

“Of note, suppression of Nphs1 and Nphs2 mRNAs was more pronounced after 4-day LMB2 treatment than 2-day treatment. During the 4-day treatment, the medium was changed, which washed away cell debris.”

To improve the logical flow based on our experimental timeline, we have relocated these two sentences to the second and third paragraphs of the Results section, respectively [Lines 174–175 and 185–187].

We hope that these revisions fully satisfy the requests of Reviewer #3 and that the manuscript is now acceptable for publication in *PLOS ONE*.

Sincerely,

Taiji Matsusaka, MD, PhD

Corresponding Author

Department of Physiology, Tokai University School of Medicine

E-mail: taijim@tokai.ac.jp
