# Supplementary material for: Podocyte injury damages podocytes in chimeric organoids
Source: PLoS One. 2026 Aug 31;21(8):e0337677. doi: 10.1371/journal.pone.0337677 (PMC13528954; doi:10.1371/journal.pone.0337677)
Supplement: Peer review history

# Peer review: Decision Letter 0

**DOI:** 10.1371/journal.pone.0337677.r001

**Authors:** Santhi Silambanan

30 Dec 2025

Dear Dr. Matsusaka,

Thank you for submitting your manuscript to PLOS ONE. After careful consideration, we feel that it has merit but does not fully meet PLOS ONE’s publication criteria as it currently stands. Therefore, we invite you to submit a revised version of the manuscript that addresses the points raised during the review process.

Please submit your revised manuscript by Feb 13 2026 11:59PM. If you will need more time than this to complete your revisions, please reply to this message or contact the journal office at plosone@plos.org. When you're ready to submit your revision, log on to https://www.editorialmanager.com/pone/ and select the 'Submissions Needing Revision' folder to locate your manuscript file.

- A letter that responds to each point raised by the academic editor and reviewer(s). You should upload this letter as a separate file labeled 'Response to Reviewers'.
- A marked-up copy of your manuscript that highlights changes made to the original version. You should upload this as a separate file labeled 'Revised Manuscript with Track Changes'.
- An unmarked version of your revised paper without tracked changes. You should upload this as a separate file labeled 'Manuscript'.

If you would like to make changes to your financial disclosure, please include your updated statement in your cover letter. Guidelines for resubmitting your figure files are available below the reviewer comments at the end of this letter.

If applicable, we recommend that you deposit your laboratory protocols in protocols.io to enhance the reproducibility of your results. Protocols.io assigns your protocol its own identifier (DOI) so that it can be cited independently in the future. For instructions see: https://journals.plos.org/plosone/s/submission-guidelines#loc-laboratory-protocols. Additionally, PLOS ONE offers an option for publishing peer-reviewed Lab Protocol articles, which describe protocols hosted on protocols.io. Read more information on sharing protocols at https://plos.org/protocols?utm_medium=editorial-email&utm_source=authorletters&utm_campaign=protocols.

We look forward to receiving your revised manuscript.

Kind regards,

Santhi Silambanan, MD, DNB

Academic Editor

PLOS One

Journal Requirements:

When submitting your revision, we need you to address these additional requirements.

1. Please ensure that your manuscript meets PLOS ONE's style requirements, including those for file naming. The PLOS ONE style templates can be found at

https://journals.plos.org/plosone/s/file?id=wjVg/PLOSOne_formatting_sample_main_body.pdf and

https://journals.plos.org/plosone/s/file?id=ba62/PLOSOne_formatting_sample_title_authors_affiliations.pdf

2. Thank you for stating the following financial disclosure:

“TM received grants from Japan Society for the Promotion of Science (Grant-in-Aid for Scientific Research, 18H02827 and 23K21437)(https://www.jsps.go.jp/english/e-grants/)”

Please state what role the funders took in the study. If the funders had no role, please state: 'The funders had no role in study design, data collection and analysis, decision to publish, or preparation of the manuscript.'

If this statement is not correct you must amend it as needed.

Please include this amended Role of Funder statement in your cover letter; we will change the online submission form on your behalf.

3. If the reviewer comments include a recommendation to cite specific previously published works, please review and evaluate these publications to determine whether they are relevant and should be cited. There is no requirement to cite these works unless the editor has indicated otherwise.

**Additional Editor Comments:**

Authors need to respond to the queries raised by reviewers.

[Note: HTML markup is below. Please do not edit.]

Reviewers' comments:

Reviewer's Responses to Questions

**Comments to the Author**

1. Is the manuscript technically sound, and do the data support the conclusions?

Reviewer #1: Yes

Reviewer #2: Yes

**********

2. Has the statistical analysis been performed appropriately and rigorously?

Reviewer #1: Yes

Reviewer #2: I Don't Know

**********

3. Have the authors made all data underlying the findings in their manuscript fully available?

The PLOS Data policy

Reviewer #1: Yes

Reviewer #2: Yes

**********

4. Is the manuscript presented in an intelligible fashion and written in standard English?

Reviewer #1: Yes

Reviewer #2: Yes

**********

Reviewer #1: The manuscript addresses an important question regarding the mechanisms by which podocyte injury propagates locally within the glomerulus. By generating chimeric kidney organoids composed of LMB2-sensitive and -resistant podocytes, the authors successfully develop an in vitro system to explore podocyte–podocyte injury transmission independent of filtration forces and other glomerular cell types. Overall, the experimental approach is technically sound, and the data support the major conclusion that direct cellular proximity is required for secondary podocyte injury

Points that require clarification or improvement:

Quantification of imaging data:

Many key observations—particularly loss of podocin or WT1 staining in indirectly injured podocytes—are presented solely as representative images. Quantitative morphometric data (e.g., podocyte counts, signal intensity, or area of staining) would significantly strengthen the conclusions.

Incomplete reporting of sample sizes in imaging experiments:

While qRT-PCR analyses include clear biological replicates, the number of organoids examined for each imaging result is not specified. This information is necessary to evaluate reproducibility.

Data availability requirements:

The manuscript states that all data are provided within the article and supporting files, but underlying raw data (e.g., Ct values for qPCR, numerical replicate values, or quantifications of immunostaining) are not included. For full compliance with PLOS data policies, these datasets should be provided as supplementary files.

Statistical analysis considerations:

The statistical methods for polysome qRT-PCR experiments are appropriate, but assumptions (e.g., normality) are not discussed. In addition, negative inhibitor experiments (gap junction inhibition, TRPC6, TGF-β, NF-κB inhibition) are evaluated qualitatively; quantitative assessment would make the interpretation more robust.

Clarity and style of writing:

The manuscript is generally well written; however, several sentences are overly long or densely structured. Minor grammatical and stylistic inconsistencies are present throughout the text, and the figure legends could be clarified further. These issues do not obscure the scientific message but should be addressed during revision.

Mechanistic implications:

While the authors appropriately acknowledge that the precise signaling mechanism remains unresolved, the Discussion could benefit from a more explicit statement that the current data rule out some pathways (e.g., GJ blockade) only partially, as the inhibitor experiments do not represent a comprehensive mechanistic screen.

Overall assessment:

The manuscript presents novel and valuable findings demonstrating that podocyte injury can propagate between directly adjacent podocytes in organoids, independently of filtration pressure or non-podocyte glomerular cells. The work is conceptually significant and suitable for publication in PLOS ONE after the concerns outlined above are addressed. The most important improvements needed relate to data quantification, data availability, and clarification of methods and writing.

Reviewer #2: The topic sounds good. The hypothesis put forward is highly relevant. The methods used are well designed. The results are well expressed and discussed.

Here are my specific comments:

* introduction

- the stage for the study is well-set by highlighting the importance of podocyte injury in glomerulosclerosis

- the authors provide a concise overview of previous studies including their previous work which helps understanding the context of this study

- please complete with the insertion of the corresponding references (ex: last sentence for the first paragraph et the second paragraph)

* methods

- methods are well-written

- please provide references for the adopted methods

- please give more details about somes methods such as the immunoprecipitation procedure and the use of the RNeasy Micro Kit.

- Can the authors present a graphical summary of the methods used? This will be very helpful in facilitating understanding for the reader.

* results

- results are well-presented in a clear and concise manner

- please identify the statistically different groups in the figures.

**********

what does this mean?). If published, this will include your full peer review and any attached files.

If you choose “no”, your identity will remain anonymous but your review may still be made public.

**Do you want your identity to be public for this peer review?**  For information about this choice, including consent withdrawal, please see our Privacy Policy

Reviewer #1: No

Reviewer #2: No

**********

[NOTE: If reviewer comments were submitted as an attachment file, they will be attached to this email and accessible via the submission site. Please log into your account, locate the manuscript record, and check for the action link "View Attachments". If this link does not appear, there are no attachment files.]

To ensure your figures meet our technical requirements, please review our figure guidelines: https://journals.plos.org/plosone/s/figures

You may also use PLOS’s free figure tool, NAAS, to help you prepare publication quality figures: https://journals.plos.org/plosone/s/figures#loc-tools-for-figure-preparation.

NAAS will assess whether your figures meet our technical requirements by comparing each figure against our figure specifications.

Attachment Submitted filename: Plos One comments Podocyte.docx

# Author comment: Author response to Decision Letter 1

**DOI:** 10.1371/journal.pone.0337677.r002

27 Jan 2026

We would like to thank the Editor and the Reviewers for their positive and encouraging evaluation of our manuscript. We appreciate the insightful and constructive comments provided, which have significantly helped us improve the quality and clarity of our work.

In response to the reviewers’ suggestions, we have performed additional experiments, including podocin and HA immunostaining, re-evaluated our statistical analyses, and provided raw data for transparency. We have also added a new supplementary figure illustrating our methodology and included additional references. The manuscript has been extensively edited for clarity, flow, and grammatical consistency.

Our point-by-point responses to the specific concerns raised by the reviewers are detailed below.

Response to Reviewer 1

1. Quantification of imaging data and sample sizes:

Many key observations—particularly loss of podocin or WT1 staining—are presented solely as representative images. Quantitative morphometric data and reporting of sample sizes are necessary.

Response: We appreciate the reviewer’s constructive suggestion. To enhance the robustness of our findings, we have now performed a quantitative analysis of podocin signal intensity for Figures 2, 5, 6, S2, S3, and S4.

To ensure accurate quantification, we addressed two technical challenges: 1) podocin intensity varies with the area of podocyte clusters within a given section, and 2) the baseline differentiation of organoids can vary between batches. To correct for these factors, we normalized the podocin-positive intensity to the HA-positive area in adjacent sections. The HA-tag, fused to a mitochondrial protein specifically in podocytes, serves as a stable internal marker because its expression is more resistant to LMB2-induced injury compared to podocyte differentiation markers. For S3 Figure, podocin intensity was normalized to the total organoid area, as NEP25 organoids lack the HA tag.

The quantitative data for each individual organoid are detailed in S2 Table, which allows for a clear assessment of the sample size for each experimental group. Typically, we analyzed n = 2-4 organoids per condition to ensure reproducibility. While the sample size for some groups was constrained by the complexity of the organoid assays, the quantitative results consistently demonstrate that podocin expression is significantly suppressed by LMB2 in both NEP25/RiboTag and chimeric organoids. Furthermore, this suppression was not attenuated by CBX or other tested inhibitors, supporting our qualitative observations. We have updated the Methods section and Figure Legends to reflect these quantitative analyses.

2. Data availability requirements:

Raw data (e.g., Ct values for qPCR, numerical replicate values, or quantifications of immunostaining) should be provided.

Response: In compliance with PLOS ONE’s data policy, we have provided all raw data. S3 Table now includes Ct values for all qPCR experiments, and S2 Table contains numerical replicate values and quantifications for all podocin immunostaining.

3. Statistical analysis considerations:

Assumptions (e.g., normality) for polysome qRT-PCR experiments are not discussed.

Response: We thank the reviewer for this important point. Since the original qPCR data exhibited a right-skewed distribution, we performed logarithmic transformations to stabilize the variance and approximate a normal distribution.

In the revised Fig. 3, we now present scatter plots with geometric means. While log-transformation was generally effective, we evaluated the normality of residuals and noted that for certain genes (e.g., Nphs1 and Wt1 in Fig. 3d), the residuals remained slightly skewed. However, alternative transformations (square-root or reciprocal) provided a marginally better fit to the normal distribution. However, the statistical significance remained unchanged regardless of the transformation method used. For instance, the adjusted P-values for Nphs1, Nphs2, Wt1, and Gadd45b remained consistently below 0.05. This confirms the robustness of our conclusions. We have added a statement regarding these transformations to the Figure 3 legend.

4. Clarity and style of writing:

Several sentences are overly long or densely structured. Minor grammatical and stylistic inconsistencies are present.

Response: We have conducted a comprehensive revision of the manuscript to improve readability. Long sentences have been shortened, and we have refined technical descriptions to be more concise. All Figure Legends have also been revised for clarity regarding experimental groups and statistical analyses.

5. Mechanistic implications:

The Discussion could benefit from a more explicit statement that the current data rule out some pathways only partially.

Response: We agree with the reviewer’s insightful point. While our inhibitor experiments (including gap junction blockade) did not show a rescue effect, we acknowledge that this does not constitute an exhaustive mechanistic screen. We have added a statement to the Discussion clarifying that these pathways are only partially ruled out and acknowledging this as a limitation of the study.

Response to Reviewer 2

1. References in Introduction:

The corresponding references should be inserted after the last sentence for the first and second paragraphs.

Response: We have now included the relevant references at the end of the first and second paragraphs of the Introduction as requested.

2. Methods details and graphical summary:

Provide more details about IP and RNeasy Micro Kit. Can the authors present a graphical summary of the methods?

Response: We have expanded the Methods section to include more detail on the immunoprecipitation procedure and the use of the RNeasy Micro Kit, including additional citations. Furthermore, we have added S7 Figure, which provides a schematic overview of the polysome analysis in chimeric organoids to facilitate reader understanding.

3. Results - Statistical identification:

Please identify the statistically different groups in the figures.

Response: We have updated Figure 3 to clearly indicate statistically significant groups using asterisks (*).

We believe that these revisions have strengthened the manuscript and addressed all the concerns raised by the reviewers. We hope the revised version is now suitable for publication in PLOS ONE.

Attachment Submitted filename: Response to Reviewers.docx

# Peer review: Decision Letter 1

**DOI:** 10.1371/journal.pone.0337677.r003

**Authors:** Santhi Silambanan

11 May 2026

Dear Dr. Matsusaka,

Thank you for submitting your manuscript to PLOS ONE. After careful consideration, we feel that it has merit but does not fully meet PLOS ONE’s publication criteria as it currently stands. Therefore, we invite you to submit a revised version of the manuscript that addresses the points raised during the review process.

Please submit your revised manuscript by Jun 25 2026 11:59PM. If you will need more time than this to complete your revisions, please reply to this message or contact the journal office at plosone@plos.org. When you're ready to submit your revision, log on to https://www.editorialmanager.com/pone/ and select the 'Submissions Needing Revision' folder to locate your manuscript file.

- A letter that responds to each point raised by the academic editor and reviewer(s). You should upload this letter as a separate file labeled 'Response to Reviewers'.
- A marked-up copy of your manuscript that highlights changes made to the original version. You should upload this as a separate file labeled 'Revised Manuscript with Track Changes'.
- An unmarked version of your revised paper without tracked changes. You should upload this as a separate file labeled 'Manuscript'.

If you would like to make changes to your financial disclosure, please include your updated statement in your cover letter. Guidelines for resubmitting your figure files are available below the reviewer comments at the end of this letter.

If applicable, we recommend that you deposit your laboratory protocols in protocols.io to enhance the reproducibility of your results. Protocols.io assigns your protocol its own identifier (DOI) so that it can be cited independently in the future. For instructions see: https://journals.plos.org/plosone/s/submission-guidelines#loc-laboratory-protocols. Additionally, PLOS ONE offers an option for publishing peer-reviewed Lab Protocol articles, which describe protocols hosted on protocols.io. Read more information on sharing protocols at https://plos.org/protocols?utm_medium=editorial-email&utm_source=authorletters&utm_campaign=protocols.

As the corresponding author, your ORCID iD is verified in the submission system and will appear in the published article. PLOS supports the use of ORCID, and we encourage all coauthors to register for an ORCID iD and use it as well. Please encourage your coauthors to verify their ORCID iD within the submission system before final acceptance, as unverified ORCID iDs will not appear in the published article. *Only*  the individual author can complete the verification step; PLOS staff *cannot*  verify ORCID iDs on behalf of authors.

We look forward to receiving your revised manuscript.

Kind regards,

Santhi Silambanan, MD, DNB

Academic Editor

PLOS One

Journal Requirements:

1. If the reviewer comments include a recommendation to cite specific previously published works, please review and evaluate these publications to determine whether they are relevant and should be cited. There is no requirement to cite these works unless the editor has indicated otherwise.

Additional Editor Comments:

The authors shall respond to the comments raised by the third reviewer.

[Note: HTML markup is below. Please do not edit.]

Reviewer's Responses to Questions

**Comments to the Author**

Reviewer #3: (No Response)

**********

2. Is the manuscript technically sound, and do the data support the conclusions?

Reviewer #3: (No Response)

**********

3. Has the statistical analysis been performed appropriately and rigorously?

Reviewer #3: (No Response)

**********

4. Have the authors made all data underlying the findings in their manuscript fully available?

The PLOS Data policy

Reviewer #3: (No Response)

**********

5. Is the manuscript presented in an intelligible fashion and written in standard English?

Reviewer #3: (No Response)

**********

Reviewer #3: The manuscript presents a well-designed experimental study investigating the propagation of podocyte injury using chimeric kidney organoids. The use of a mosaic organoid system to isolate direct cell–cell interactions is a notable strength, and the findings provide meaningful insight into mechanisms underlying local injury spread in kidney disease. The combination of imaging, molecular analysis, and functional assays supports the conclusions. Overall, the study is of good quality, and only minor revisions are required to further improve clarity and presentation.

1. Some sentences, particularly in the introduction and discussion sections, are slightly long and could be simplified to improve readability.

2. The distinction between “indirect injury,” “secondary injury,” and “bystander effect” could be clarified and used more consistently throughout the manuscript.

3. The novelty of the study could be stated more concisely, especially in relation to previous in vivo findings mentioned in the introduction.

4. The description of the organoid model and its limitations (e.g., absence of endothelial and mesangial cells) could be discussed a bit more clearly.

5. Figure captions (e.g., Figs. 2 and 5) could be slightly expanded to better guide the reader in interpreting key findings such as the loss of podocin in non-targeted cells.

6. The results section would benefit from a bit more explanation of the biological reasoning behind the observed trends.

7. The discussion of possible mechanisms (e.g., gap junctions, TNTs) could be presented more cautiously as potential explanations rather than definitive conclusions.

8. Minor typographical and formatting inconsistencies (e.g., spacing, notation, gene names) should be carefully revised.

**********

what does this mean?). If published, this will include your full peer review and any attached files.

If you choose “no”, your identity will remain anonymous but your review may still be made public.

**Do you want your identity to be public for this peer review?**  For information about this choice, including consent withdrawal, please see our Privacy Policy

Reviewer #3: No

**********

[NOTE: If reviewer comments were submitted as an attachment file, they will be attached to this email and accessible via the submission site. Please log into your account, locate the manuscript record, and check for the action link "View Attachments". If this link does not appear, there are no attachment files.]

To ensure your figures meet our technical requirements, please review our figure guidelines: https://journals.plos.org/plosone/s/figures

You may also use PLOS’s free figure tool, NAAS, to help you prepare publication quality figures: https://journals.plos.org/plosone/s/figures#loc-tools-for-figure-preparation.

NAAS will assess whether your figures meet our technical requirements by comparing each figure against our figure specifications.

Attachment Submitted filename: PONE 1 3rd comments.docx

# Author comment: Author response to Decision Letter 2

**DOI:** 10.1371/journal.pone.0337677.r004

29 May 2026

May 28, 2026

Dear Editor and Reviewers,

We would like to express our sincere gratitude to the reviewers for their constructive comments and highly positive evaluation of our manuscript entitled "Podocyte injury damages podocytes in chimeric organoids" (Manuscript ID: PONE-D-25-60672R).

We have carefully addressed all the points raised by Reviewer #3. The manuscript has been revised accordingly, and we believe these changes have significantly improved the clarity and quality of our manuscript.

Response to Academic Editor

Journal Requirement / Recommendation: If applicable, we recommend that you deposit your laboratory protocols in protocols.io to enhance the reproducibility of your results. Protocols.io assigns your protocol its own identifier (DOI) so that it can be cited independently in the future.

Response: We sincerely appreciate the editor’s valuable recommendation to utilize protocols.io to enhance reproducibility. Regarding the protocol for generating the kidney organoids, which is a key characteristic of our study, we would like to clarify that the detailed step-by-step procedures have already been fully established and previously published by our co-authors (referred to as Ref. 18 in the manuscript). Because this robust protocol is already available to the public and properly cited in our Methods section, we have not deposited it as a new protocol on protocols.io. We hope the editor finds this explanation acceptable, as the reproducibility of the method is already well-secured through our previous publication.

Below is our point-by-point response to the reviewer’s comments.

Response to Reviewer #3

General Comment:

The manuscript presents a well-designed experimental study investigating the propagation of podocyte injury using chimeric kidney organoids. The use of a mosaic organoid system to isolate direct cell–cell interactions is a notable strength, and the findings provide meaningful insight into mechanisms underlying local injury spread in kidney disease. The combination of imaging, molecular analysis, and functional assays supports the conclusions. Overall, the study is of good quality, and only minor revisions are required to further improve clarity and presentation.

Response: We sincerely thank the reviewer for the highly encouraging feedback and appreciation of our mosaic organoid system. We agree that the suggested minor revisions greatly enhance the clarity and presentation of our findings.

Comment 1: Some sentences, particularly in the introduction and discussion sections, are slightly long and could be simplified to improve readability.

Response: We agree with the reviewer's assessment. We have simplified several long and complex sentences throughout the Introduction and Discussion sections to enhance readability.

• In the Introduction [Lines 50-52]: We split the sentence regarding LMB2 clearance into two separate sentences:

o Old: "Notably, a single injection of LMB2 results in progressive kidney injury over several weeks, even though the toxin is rapidly cleared and podocytes with activated caspase are eliminated within seven days."

o Revised: "Notably, a single injection of LMB2 results in progressive kidney injury extending over several weeks. This progression occurs despite the rapid clearance of the toxin and the elimination of caspase-activated podocytes within seven days."

• In the Discussion [Lines 280-282]: We similarly simplified the sentence describing injury propagation:

o Old: "Our findings indicate that podocytes directly injured by LMB2 can induce secondary damage to neighboring, initially intact podocytes within chimeric organoids, despite the absence of glomerular filtration or other glomerular cell types."

o Revised: "Our findings indicate that podocytes directly injured by LMB2 induce indirect damage to neighboring, initially intact podocytes within chimeric organoids. Importantly, this propagation occurs even in the absence of glomerular filtration or other glomerular cell types."

Comment 2: The distinction between “indirect injury,” “secondary injury,” and “bystander effect” could be clarified and used more consistently throughout the manuscript.

Response: Thank you for this excellent suggestion. To ensure conceptual consistency, we have standardized our terminology by shifting our preference to the term "indirect injury" throughout the entire manuscript, including the Abstract, Results, Discussion, and Figure Legends.

Comment 3: The novelty of the study could be stated more concisely, especially in relation to previous in vivo findings mentioned in the introduction.

Response: We have revised the summary of our findings at the end of the Introduction to state the novelty of this study more concisely and clearly. We emphasized that our organoid model successfully isolates direct cell-cell interactions from confounding in vivo environmental factors.

• Revised text [Lines 74-77]: "Using this new in vitro system, we successfully isolated direct podocyte-to-podocyte communication from confounding in vivo factors, such as filtration pressure and other types of cells. We demonstrate for the first time in vitro that podocyte injury propagation is an autonomous, self-sustaining process driven by direct podocyte-to-podocyte interactions."

Comment 4: The description of the organoid model and its limitations (e.g., absence of endothelial and mesangial cells) could be discussed a bit more clearly.

Response: As suggested, we have expanded the "Limitations" section in the Discussion to provide a more comprehensive and rigorous description of our organoid model. Specifically, we have explicitly stated that our organoids are derived exclusively from nephron progenitor cells (thereby lacking interstitial and ureteric bud derivatives) and have discussed how the absence of endothelial and mesangial cells represents a simplified but advantageous system that still warrants cautious interpretation regarding native diseases.

• Revised text [Lines 274-279]: "Third, our organoids are generated exclusively from nephron progenitor cells, thereby lacking components derived from interstitial progenitors and ureteric buds. Consequently, the tissue architecture of these organoids differs from that of the in vivo counterpart. While the absence of endothelial and mesangial cells allowed us to isolate and prove that they are not required for injury propagation, in native glomerular diseases, these cell types likely modulate the autonomous podocyte pathways described here."

Comment 5: Figure captions (e.g., Figs. 2 and 5) could be slightly expanded to better guide the reader in interpreting key findings such as the loss of podocin in non-targeted cells.

Response: We have expanded the legends for Figures 2 and 5 to better guide the reader in interpreting the cell-type specificity and the visual evidence of indirect injury propagation.

• Revised Fig. 2 Legend [Lines 420-421]: "...LMB2 treatment causes the loss of cluster morphology and podocyte marker expression, whereas proximal tubule structures (LTL-positive) remain unaffected, demonstrating the cell-type specificity of the immunotoxin."

• Revised Fig. 5 Legend [Lines 441-442]: "Note the complete loss of hCD25(+) podocytes (d) and the indirect reduction of podocin staining (f) in the persisting HA(+) podocytes (e), visually demonstrating the propagation of injury to non-targeted neighboring cells within the same glomerular-like structure."

Comment 6: The results section would benefit from a bit more explanation of the biological reasoning behind the observed trends.

Response: We agree with the reviewer's comment. To provide a clearer biological context for the observed gene expression trends, we have added a reference to previous in vivo findings, clarifying that the expression changes in our organoid model closely mirror those observed in chimeric mice. Additionally, we have explicitly stated the biological reasoning for targeting gap junctions in our inhibitor experiments.

The Results section has been revised as follows:

• In the Results section [Lines 202-203]: We added a clause to contextualize the gene expression changes with our previous in vivo data:

o Old: "Following LMB2 treatment, these indirectly affected podocytes displayed reduced Nphs1, Nphs2, and Wt1 expression levels and increased Gadd45b expression level."

o Revised: "Following LMB2 treatment, these indirectly affected podocytes displayed reduced Nphs1, Nphs2, and Wt1 expression levels and increased Gadd45b expression level, which is consistent with indirectly injured podocyte profiles in chimeric mice [13]."

• In the Results section [Lines 203-204]: We provided the biological rationale for examining gap junctions in the context of indirect injury propagation:

o Added text: Because it is frequently reported that gap junctions mediate bystander killing in other tissues by transferring cytotoxic molecules, we hypothesized that intercellular signaling through gap junctions might mediate the indirect injury."

Comment 7: The discussion of possible mechanisms (e.g., gap junctions, TNTs) could be presented more cautiously as potential explanations rather than definitive conclusions.

Response: We agree with the reviewer and have softened our tone in the Discussion section to present gap junctions and tunneling nanotubes (TNTs) more cautiously as potential hypotheses rather than definitive conclusions.

• Revised text for Gap Junctions [Lines 249-252]: "While these pharmacological results suggest that gap junctions may not be the principal mediators of bystander injury in this model, further genetic validation would be required to definitively exclude their contribution."

• Revised text for TNTs [Lines 263-268]: " Under such conditions, it is conceivable that injury signals may be transmitted from hCD25-positive to hCD25-negative podocytes via TNTs." …. from mosaic mice (S6 Figure). “Direct assessment of TNT involvement remains an objective for future studies.”

Comment 8: Minor typographical and formatting inconsistencies (e.g., spacing, notation, gene names) should be carefully revised.

Response: We have thoroughly reviewed the entire manuscript and corrected all typographical errors and formatting inconsistencies. Specifically, we have:

1. Corrected the gene name typo from Npsh2 to Nphs2 [Line 185].

2. Standardized the hyphenation for "2-day treatment" to match "4-day LMB2 treatment" [Line 186].

3. Unified the style for supplemental figure citations to (S2 Figure) throughout the text [Line 179].

4. Unified the style of unit (mL).

5. Corrected the spacing around the statistical symbols (e.g., p-value < 0.05) [Line 151].

6. Corrected spelling of “Schema” [Line 461].

Additional Revision by the Authors: While revising the manuscript, we noticed that the placement of the following sentences was inappropriate, given that most of the inhibitor experiments were performed using the 2-day LMB2 treatment protocol:

“Of note, suppression of Nphs1 and Nphs2 mRNAs was more pronounced after 4-day LMB2 treatment than 2-day treatment. During the 4-day treatment, the medium was changed, which washed away cell debris.”

To improve the logical flow based on our experimental timeline, we have relocated these two sentences to the second and third paragraphs of the Results section, respectively [Lines 174–175 and 185–187].

We hope that these revisions fully satisfy the requests of Reviewer #3 and that the manuscript is now acceptable for publication in PLOS ONE.

Sincerely,

Taiji Matsusaka, MD, PhD

Corresponding Author

Department of Physiology, Tokai University School of Medicine

E-mail: taijim@tokai.ac.jp

Attachment Submitted filename: Response to Reviewers-2.docx

# Peer review: Decision Letter 2

**DOI:** 10.1371/journal.pone.0337677.r005

**Authors:** Santhi Silambanan

17 Aug 2026

<p>Podocyte injury damages podocytes in chimeric organoids

PONE-D-25-60672R2

Dear Dr. Matsusaka,

We’re pleased to inform you that your manuscript has been judged scientifically suitable for publication and will be formally accepted for publication once it meets all outstanding technical requirements.

Within one week, you’ll receive an e-mail detailing the required amendments. When these have been addressed, you’ll receive a formal acceptance letter and your manuscript will be scheduled for publication.

An invoice will be generated when your article is formally accepted. Please note, if your institution has a publishing partnership with PLOS and your article meets the relevant criteria, all or part of your publication costs will be covered. Please make sure your user information is up-to-date by logging into Editorial Manager at Editorial Manager® and clicking the ‘Update My Information' link at the top of the page. For questions related to billing, please contact billing support.

If your institution or institutions have a press office, please notify them about your upcoming paper to help maximize its impact. If they’ll be preparing press materials, please inform our press team as soon as possible -- no later than 48 hours after receiving the formal acceptance. Your manuscript will remain under strict press embargo until 2 pm Eastern Time on the date of publication. For more information, please contact onepress@plos.org.

Kind regards,

Santhi Silambanan, MD, DNB

Academic Editor

PLOS One

# Editor report: Acceptance letter

**DOI:** 10.1371/journal.pone.0337677.r006

**Authors:** Santhi Silambanan

PONE-D-25-60672R2

PLOS One

Dear Dr. Matsusaka,

I'm pleased to inform you that your manuscript has been deemed suitable for publication in PLOS One. Congratulations! Your manuscript is now being handed over to our production team.

At this stage, our production department will prepare your paper for publication. This includes ensuring the following:

* All references, tables, and figures are properly cited

* All relevant supporting information is included in the manuscript submission,

* There are no issues that prevent the paper from being properly typeset

You will receive further instructions from the production team, including instructions on how to review your proof when it is ready. Please keep in mind that we are working through a large volume of accepted articles, so please give us a few days to review your paper and let you know the next and final steps.

Lastly, if your institution or institutions have a press office, please let them know about your upcoming paper now to help maximize its impact. If they'll be preparing press materials, please inform our press team within the next 48 hours. Your manuscript will remain under strict press embargo until 2 pm Eastern Time on the date of publication. For more information, please contact onepress@plos.org.

You will receive an invoice from PLOS for your publication fee after your manuscript has reached the completed accept phase. If you receive an email requesting payment before acceptance or for any other service, this may be a phishing scheme. Learn how to identify phishing emails and protect your accounts at https://explore.plos.org/phishing.

If we can help with anything else, please email us at customercare@plos.org.

Thank you for submitting your work to PLOS One and supporting open access.

Kind regards,

PLOS One Editorial Office Staff

on behalf of

Dr. Santhi Silambanan

Academic Editor

PLOS One
